# Green Foxtail-Derived Endophytic Fungus *Chaetomium novozelandicum* SV7 Produced Orsellinic Acid Ester Glycosides Active Against Rice Blast Pathogen

**DOI:** 10.3390/ijms27198582

**Published:** 2026-09-25

**Authors:** Jianing Wang, Yonglin Yang, Jinlan He, Yanxue Peng, Yifei Qin, Xuan Zhang, Zhen Shen, Jun Yang, Ligang Zhou, Daowan Lai

**Affiliations:** 1 Sanya Institute, China Agricultural University, Sanya 572025, Chinayangj@cau.edu.cn (J.Y.); 2 State Key Laboratory of Agricultural and Forestry Biosecurity, MARA Key Lab of Pest Monitoring and Green Management, College of Plant Protection, China Agricultural University, Beijing 100193, Chinalgzhou@cau.edu.cn (L.Z.)

**Keywords:** plant–microbe interaction, *Setaria viridis*, endophytic fungi, *Magnaporthe oryzae*, *Chaetomium novozelandicum*, antifungal activity, natural products, structure elucidation, orsellinic acid ester, plant infection assay

## Abstract

Plants encounter various fungi during their life history, with some of them pathogenic, some beneficial, and some neither, forming complex plant–fungi interactions. Endophytic fungi are a type of fungi that live inside the plant, and are often beneficial to the host. In this study, the endophytic fungi of green foxtail (*Setaria viridis*) were explored for their defensive role against the pathogenic fungus *Magnaporthe oryzae*, which causes rice blast, one of the most devastating crop diseases. The endophytic fungi were isolated, taxonomically identified, fermented, and chemically extracted. The fungal extracts were screened for their inhibition against *M. oryzae* using mycelial growth rate assay, of these, *Chaetomium novozelandicum* SV7 displayed the most inhibition. A scale-up fermentation of this fungus, followed by extraction and chromatographic purification, led to the isolation and identification of eleven orsellinate-types of metabolites (**1**–**11**), including four new compounds (**1**, **2**, **6**, **8**), named chaetorsellide A-D. The structures of the new compounds were elucidated by means of NMR, HRMS, and quantum chemical calculations. All these metabolites were reported from *C. novozelandicum* for the first time, with five of them being orsellinic acid ester glycosides (**1**–**5**). Plant infection assay was carried out for the isolated compounds, among which compounds **8** and **3** displayed significant activities, with **8** being the most potent. These results not only reveal that these compounds have potential to be developed as natural fungicides against the rice blast pathogen, but also add one more example for exploring new antimicrobial substances from plant–microbe interactions.

## 1. Introduction

Plants harbor a variety of fungi, especially those colonizing internal plant tissues, called endophytic fungi, which cause no apparent symptoms to the host. Endophytes have adapted to the plant-cell environment through co-evolution, and obtained nutrients and support from the plant; on the other hand, endophytes can enhance the resistance and resilience of the plant against abiotic and biotic stresses, such as pathogen infection and salt stress [1,2]. Searching for specific endophytes that mediate defense against pathogens is an interesting topic in plant–microbe interaction research. These not only advance our knowledge of the endophyte–plant interactions, but also contribute to plant protection. In addition, new anti-microbial natural products have been reported to originate from such interactions to a large extent [3].

As a continued interest in searching for new bioactive substances from endophytic fungi derived from plants [4,5], we focused on the endophytes of green foxtail (*Setaria viridis* (L.) Beauv.) in this study. *S. viridis* is an important model plant for the study of C4 photosynthesis in Panicoideae grasses [6]. Recently, the ethanol extract of *S. viridis* was found to counter obesity-induced muscle atrophy and metabolic dysfunction in high-fat diet-induced obese mice, supporting its potential as an effective nutritional strategy for managing sarcopenic obesity [7].

This grass is commonly seen as a weed in the paddy field, where rice plants are cultivated. Throughout the life cycle, rice suffers from many diseases. Among them, rice blast is one of the most destructive, which is caused by *Magnaporthe oryzae* (anamorph: *Pyricularia oryzae*), an Ascomycota fungal pathogen, causing 5–30% yield losses worldwide [8]. It has been reported that *M. oryzae* isolated from rice could infect green foxtail, which also belongs to Gramineae like rice [9]. Despite that, it is commonly seen that the weedy grass is healthy without apparent symptoms on it. We hypothesized that the endophytes inside the plant could help the host defend against the pathogen *M. oryzae*. It is intriguing to isolate and identify the specific endophytes that exert such roles.

Hence, a series of experiments were designed and carried out as depicted in Figure 1. First, the healthy plants were collected; then, the endophytic fungi were isolated, and identified taxonomically. After that, each fungus was cultivated in a small-scale and the biomass were extracted with organic solvent (EtOAc) to obtain the crude extract, which was subsequently subjected to mycelial growth rate assay with *M. oryzae* as the tested pathogen. The inhibitory rates of the fungal extracts against the growth of the pathogen were recorded. Of these, the endophyte (SV7) displayed the highest inhibition and was selected for further study.

Chemical investigations of this fungus were carried out, resulting in the isolation and identification of eleven orsellinic acid esters (**1**–**11**), including four new compounds, and five orsellinic acid ester glycosides. These compounds were evaluated for their inhibition against the pathogen *M. oryzae* using plant infection assay. The isolation, purification, and structural elucidation of the new fungal metabolites, as well as their bioactivities are reported in detail.

## 2. Results

### 2.1. Endophytic Fungi from S. viridis Showed Inhibition Against Rice Blast Pathogen

#### 2.1.1. Endophytic Fungi Isolation, and Identification

Healthy *S. viridis* was disinfected, and dissected before being inoculated onto the PDA (potato dextrose agar) plate, and kept in the dark at 28 °C. The plate was checked daily for the appearance of fungal hyphae, which were then transferred to a new PDA plate. The fungal isolation and purification were continued, until no new hyphae were observed. This resulted in a total of six different fungal specimens after taxonomical identification via morphological and molecular examination with ITS sequencing (Figure 1A).

#### 2.1.2. Evaluating the Antifungal Activities Using Mycelial Growth Rate Assay

Then, a small scale fermentation of these fungi was carried out in rice medium (30 g) for 15 days, before harvest and extraction with EtOAc. The obtained fungal extracts were evaluated for their inhibition against the rice blast pathogen *M. oryzae* using the mycelial growth rate method. Briefly, the extract was dissolved in DMSO and mixed with the molten agar before plating, then the pathogen was inoculated onto the middle of the solidified agar plate. The growth of the mycelia (diameter) was recorded daily until the pathogen on the blank control (DMSO) reached the edge of the plate. The inhibition rate was calculated by comparison with the blank control. In this study, each fungal extract was tested (Figure 1A). As a result, five of the fungal extracts displayed inhibition against the mycelia growth of *M. oryzae*, with SV7 showing the best inhibition, with an inhibition rate of 45.74%, though not as active as the positive control (Figure S1b, see the supporting information). Meanwhile, in a dual-culture assay, SV7 exhibited a prominent inhibitory effect against *M*. *oryzae*, with an inhibition rate of 48.33% (Figure S1a). Hence, this fungus (SV7) was selected for further chemical investigation. It was identified as *Chaetomium novozelandicum* by morphological examination and ITS sequence comparison (Figure 1B).

### 2.2. Compound Identification from C. novozelandicum SV7

The endophytic fungus was fermented on a large scale for a month on rice media, followed by extraction with EtOAc and defatted with petroleum ether to give the crude extract. A detailed chromatographic isolation of this extract using reverse-phase chromatography over ODS, size-exclusion chromatography over Sephadex LH-20, and purification using semi-preparative HPLC, resulted in eleven orsellinate derivatives, including four new (**1**–**2**, **6**, and **8**) and seven (**3**–**5**, **7**, **9**–**11**) known compounds. Among them, five were orsellinic acid ester glycosides (**1**–**5**) (Figure 2). The known compounds were identified as orsellides C (**3**), A (**4**), and B (**5**) [10], globosumone B (**7**) [11], (1*R*,2*S*)-1-carboxy-2-hydroxypropyl 2,4-dihydroxy-6-methylbenzoate (**9**) [12], elatumone B (**10**) [13], and methyl orsellinate (**11**) [14], by comparison of their spectroscopic data with the literature. The new compounds were elucidated by a combined analysis of the 1D, 2D NMR spectra and HRMS data (*vide infra*).

#### 2.2.1. Structural Elucidation of Chaetorsellide A (**1**)

Compound **1** was isolated as a light-yellowish amorphous powder, whose molecular formula was determined as C_14_H_18_O_6_ by analysis of the HRESIMS spectrum (Figure S4-7, see the Supplementary Information), in which a prominent deprotonated pseudomolecular peak was observed at *m*/*z* 281.1012 (calc. for C_14_H_17_O_6_^−^, 281.1031).

Inspection of its ^1^H NMR spectrum (Figure S4-1) (Table 1) suggested the presence of two meta-coupling aromatic protons at *δ*_H_ 6.16, 6.21 (each d, *J* = 2.5 Hz), indicating the existence of a tetra-substituted benzene ring. In addition, several oxymethine, oxymethylene, and aliphatic methylene protons, as well as an aromatic methyl group (*δ*_H_ 2.52, s), and a doublet methyl group (*δ*_H_ 1.21, d) were seen in the ^1^H NMR spectrum. Checking the ^13^C NMR spectrum (Figure S4-2) (Table 1) revealed the presence of fourteen carbon signals, which were ascribed to an ester carbonyl (*δ*_C_ 172.6), six sp^2^-hybridized carbons, which included two methine (*δ*_C_ 101.7/112.6), and four quaternary ones with two being oxygenated (*δ*_C_ 166.3/163.9), in addition to three oxygenated methine (*δ*_C_ 69.9/77.2/73.6), one oxygenated methylene (*δ*_C_ 71.3), an aliphatic methylene (*δ*_C_ 39.5), and two methyl groups (*δ*_C_ 24.6/21.4) in the upfield region, with the aid of the HSQC experiment (Figure S4-3). Obviously, these six sp^2^-hybridized carbons constitute a tetra-substituted benzene ring, which is consistent with its UV spectrum showing maximum absorptions at 199, 215, 263, 298 nm (Figure S4-6).

Analysis of the HMBC spectrum (Figure S4-4) revealed the presence of an orsellinic acid unit, as correlations were found from the aromatic methyl group (*δ*_H_ 2.52, Me-8) to C-1 (*δ*_C_ 105.9), C-6 (*δ*_C_ 144.8), C-5 (*δ*_C_ 112.6), and the ester carbonyl (C-7, *δ*_C_ 172.6, via a long-range correlation), from aromatic proton H-3 (*δ*_H_ 6.16) to C-1, C-2 (*δ*_C_ 166.3), and C-4 (*δ*_C_ 163.9), and from H-5 (*δ*_H_ 6.21) to C-1, C-4, and Me-8 (*δ*_C_ 24.6) (Figure 3A). This unit accounts for five degrees of unsaturation, thus the remaining signals (six carbons) have to constitute a ring to fulfill the six degrees of unsaturation required by the molecular formula.

The remaining six carbons with four being oxygenated were reminiscent of a deoxyhexose, whose connectivity was established by detailed examination of the HMBC correlations (Figure 3A). Starting from the terminal methyl group (*δ*_H_ 1.21, Me-6′), which showed HMBC correlations to the oxygenated methine (*δ*_C_ 73.6, CH-5′), and a methylene (*δ*_C_ 39.5, CH2-4′); this methylene (*δ*_H_ 2.25, 1.44) in turn showed correlations to two consecutive oxygenated methines (*δ*_C_ 77.2, 69.9) that were assigned to CH-3′, and CH-2′, respectively. The remaining oxymethylene (*δ*_H_ 3.96, 3.25/*δ*_C_ 71.3) has to locate at C-1′ to complete the six-membered hexose ring, as it displayed correlations to CH-2′, CH-3′, and CH-5′ (Figure 3A). Finally, the significant downfield shift (*δ*_C_ 77.2, *δ*_H_ 5.06) of CH-3′ indicated it was esterified, which was confirmed by the HMBC correlation seen from H-3′ to the ester carbonyl C-7. Hence, the gross structure of **1** was established as an orsellinic acid ester glycoside.

The relative configuration of the sugar part was determined by analysis of the NOESY spectrum (Figure S4-5) and the ^1^H-^1^H coupling constants (Table 1). The large *J* values of H-4′b (*δ*_H_ 1.44) between vicinal protons (11.3 Hz) indicating it was located at axial position with regard to the chair-conformation of the six-membered ring. The NOESY correlation between H-4′ax (*δ*_H_ 1.44), Me-6′ (*δ*_H_ 1.21), and H-2′ (*δ*_H_ 3.74) suggested these protons were located at the same face (tentatively β) (Figure 3B), while the correlations between H-4′eq (*δ*_H_ 2.25), H-3′ (*δ*_H_ 5.06), H-5′ (*δ*_H_ 3.63), and H-1′b (*δ*_H_ 3.25) revealed these protons were oriented to the opposite face.

The absolute configuration of **1** was determined by comparison of the optical rotatory value with that of the known analogs (Figure 3C). The optical rotation for **1** was +60, while the synthetic sugar derivative **1m** with 2′*S*, 3′*S*, 5′*R*-configuration was also positive ([α]^25^_D_ (*c* = 1.0, CHCl_3_) = +12.98) [15]; in contrast, the compound (**1n**) with a mirror-imaged stereochemistry displayed a negative optical rotation ([α]^23^_D_ (*c* = 1.0, CHCl_3_) = −34.3) [16]. Hence, the absolute configuration of **1** was deduced as 2′*S*, 3′*S*, 5′*R*. This compound was identified a new compound, for which a trivial name chaetorsellide A was designated.

#### 2.2.2. Structural Elucidation of Chaetorsellide B (**2**)

Compound **2** was also isolated as a pale-yellowish compound, which showed a similar UV absorption (Figure S5-6) as that of **1**, indicating a congener of **1**. Its molecular formula was determined as C_14_H_16_O_6_ by HRESIMS (Figure S5-7), which contained two less hydrogen atoms than that of **1**.

Analysis of the ^1^H, ^13^C NMR data (Table 1, Figure S5-(1,2)) revealed the presence of an orsellinic acid moiety and a deoxyhexose unit as well. The notable difference was that the downfield-shifted oxymethine observed in **1** had disappeared in **2**; instead, one oxygenated quaternary carbon (*δ*_C_ 105.03) was seen. Then, the HSQC (Figure S5-3) and HMBC (Figure S5-4) experiments were used to determine the gross structure of **2**. The HMBC correlations from the oxymethylene (CH_2_-1′, *δ*_H_ 3.88/3.63) to CH-2′ (*δ*_C_ 70.9), and the oxygenated quaternary carbon (*δ*_C_ 105.03), as well as from methylene (CH_2_-4′, *δ*_H_ 2.40/1.58) to CH-2′ and this quaternary carbon, allowing its location at C-3′ (Figure 4A). Moreover, considering the large ^13^C NMR chemical shift for C-3′, it has to be dioxygenated.

By comparing the NMR data with that of **1**, the ester linkage was deduced to be retained at C-3′ in **2**, as the alternative esterification at C-2′ was ruled out, since no obvious ^1^H downfield shift was observed for H-2′. To fulfill the molecular formula, one additional ring (ether ring) had to be present in **2**. For this purpose, an ether linkage between C-3′ and C-2 of the benzene ring was put forth. The alternative ether linkage between C-2′ and C-2 was ruled out due to lack of HMBC correlation from H-2′ to C-2, and a much higher energy than the former proposed structure as predicted by molecular modeling. Hence, a tricyclic skeleton of **2** was proposed, featuring a novel 4*H*-benzo[*d*][1,3]dioxin-4-one spirally fused to a tetrahydropyran ring.

The relative configuration of **2** was determined by analysis of the NOESY spectrum (Figure S5-5). As shown in Figure 4B, the NOESY correlation between H-4′ax (*δ*_H_ 1.58), Me-6′ (*δ*_H_ 1.11), and H-2′ (*δ*_H_ 3.84) suggested these protons were located at β-face, while the correlations between H-4′eq (*δ*_H_ 2.40), H-5′ (*δ*_H_ 3.69), and H-1′b (*δ*_H_ 3.63) revealed these protons were oriented to the α-face. Meanwhile, the correlations between H-3 (*δ*_H_ 6.34), H-1′b, H-5′, and H-4′eq revealed that the ester bond was placed equatorially.

A detailed comparison of the NMR data (Table 1), revealed that the ester carbonyl (C-7, *δ*_C_ 162.0) in **2** was shifted upfield by 10.6 ppm comparing to that of **1**. To confirm this assignment, we carried out a ^13^C NMR calculation, which is a robust tool in structure determination [17,18].

Briefly, the conformational searches were carried out using Merck Molecular Force Field. Then, the geometry optimization of these conformers at the B3LYP/6-31+G(d,p) level resulted in eight conformers with population (≥1%), which all had a chair conformation for the sugar part, with the ester bond being equatorial while the ether bond was axial, but differing only at the orientations of the hydrogen of 2′-OH and 4-OH (Figure S3). The subsequent ^13^C NMR calculations for these conformers were performed at the level of mPW1PW91/6-311+G(2d,p), and the resulting data are summarized in Table 2. It can be seen from the table, the calculated chemical shifts reproduced the experimental data well, with average absolute difference (δcal-δexp) 1.43 ppm, and RMSD 1.69 ppm. Particularly, the calculated chemical shift for the ester carbonyl is 160.26, which supported our assignment. These data also confirmed the proposed stereochemistry of **2**.

To determine the absolute configuration, the TD-DFT ECD computations were performed. The optimized conformers with population (≥1%) mentioned above were subjected to ECD calculation at the PBE0/TZVP level (PCM=MeOH). The resulting spectrum was Boltzmann-averaged, and compared with the experimental one. As shown in Figure 4C, the calculated spectrum matched the experimental one. Therefore, the absolute configuration of **2** was determined as 2′*S*, 3′*S*, 5′*R*, as shown in Figure 2. This was a new compound, and named chaetorsellide B.

#### 2.2.3. Structural Elucidation of Chaetorsellide C (**6**)

Chaetorsellide C (**6**) was characterized as a pale-yellowish compound, and its molecular formula was established as C_13_H_18_O_6_ by analysis of the HRESIMS spectrum (Figure S6-6). The UV spectrum (Figure S6-5) indicated it was an analog of compound **1**, due to the presence of an orsellinic acid unit. However, the molecular formula of **6** is one carbon less than that of **1**, and its proton-deficiency index is five, revealing that the sugar part was a pentose and in a linear form (not a cyclic hexose, as in **1**).

Detailed analysis of the NMR data (Table 3, and Figure S6-(1–4)) was carried out to elucidate the structure. The HMBC correlations from the terminal methyl group (Me-5′, *δ*_H_ 1.21, d/*δ*_C_ 23.6) to one oxymethine (CH-4′, *δ*_H_ 4.00/*δ*_C_ 66.7), and one aliphatic methylene (CH_2_-3′, *δ*_H_ 1.73, 1.66/*δ*_C_ 43.4) were seen, which in turn correlated to one additional oxymethine (CH-2′, *δ*_H_ 4.10/*δ*_C_ 68.9), and one oxymethylene (CH_2_-1′, *δ*_H_ 4.35, 4.27/*δ*_C_ 69.7) (Figure 5A). Finally, the correlation from this oxymethylene to the ester carbonyl (C-7, *δ*_C_ 172.8) complete the connectivity between the orsellinic acid and the side chain, hence the gross structure of **6**.

The absolute configuration of **6**, having a chiral 2′,4′-diol, was determined by measuring the CD spectrum of its *in situ* formed [Mo_2_(OAc)_4_] complex [19]. The CD spectra were recorded after addition of [Mo_2_(OAc)_4_] to the DMSO solution of **6**. As shown in Figure 6A, a clear Mo-induced CD spectrum was seen after subtracting its own CD spectrum (**6** itself). This implied a predominant *syn*-configuration of the 2′,4′-diol, i.e., **6a** or **6b** (Figure 6B), as otherwise, no CD spectrum would be observed [19]. Furthermore, the positive cotton effect in the range of 380–450 nm (band II), indicated the absolute configuration was 2′*R*, 4′*R*, as depicted in **6a** (Figure 6B).

#### 2.2.4. Structural Elucidation of Chaetorsellide D (**8**)

Chaetorsellide D (**8**) was determined to have a molecular formula of C_11_H_12_O_6_ via HRESIMS analysis (Figure S7-5), and its UV spectrum (Figure S7-4) resembled those of the co-isolated compounds, indicating it was also an orsellinate.

Scrutiny of its NMR data (Table 3, Figure S7-(1–3)) revealed the presence of an orsellinic acid unit, in addition to three carbon signals unassigned. The connectivity of these moieties were finished by analysis of the HMBC spectrum (Figure 5B and Figure S7-3). The correlation from the methoxyl group (2′-OMe, *δ*_H_ 3.79/*δ*_C_ 52.8) to one ester carbonyl (C-2′, *δ*_C_ 170.0), as well as from the oxymethylene (CH_2_-1′, *δ*_H_ 4.91/*δ*_C_ 61.7) to this carbonyl, and also the orsellinic carbonyl (C-7, *δ*_C_ 171.9), revealed the presence of two ester moieties bridged by a methylene group (CH_2_-1′). Hence, compound **8** was elucidated as shown in Figure 2.

### 2.3. Antifungal Activities of the Isolated Compounds Against the Rice Blast Pathogen Using Plant Infection Assay

The plant infection assays for the isolated compounds were performed on seedling leaves of barley. For each compound, two final concentrations (100 and 25 μg/mL) were tested, with 1% DMSO as the blank solvent control, with the commercial fungicide pyraclostrobin (Pyr) as the positive control. The results for all the isolated compounds are shown in Table 4 and Figure S2, among which only compounds **8** and **3** displayed significant inhibition against the plant infection. As shown in Figure 7A,B, compound **8** displayed 100% inhibition at 100 μg/mL, with no lesions observed, similar to the PYR treatment, while compound **3** showed 80.5% inhibition at the same concentration judging from the lesion area. When tested at 25 μg/mL, inhibitory rates of 57.6%, and 44.1%, were recorded for **8** and **3**, respectively (Table 4).

Next, the most potent compound (i.e., **8**) was re-assayed on rice leaves at three different concentrations (100, 50, and 25 μg/mL). As can be seen from Figure 7C,D, all the tested concentrations displayed significant inhibition comparing to the control, with inhibition rates of 85.8%, 67.5%, and 56.6%, respectively.

## 3. Discussion

*S. viridis* is often studied as a model grass for herbicidal investigation in the literature, while few studies have been made of its endophytic fungi. The only example was *Edenia gomezpompae* SV2, isolated from the stem of *S. viridis*, which was capable of producing preussomerins [20]. In this study, the endophytic *C. novozelandicum* SV7 was found to produce various orsellinic acid esters, which were isolated from this species for the first time.

Simple orsellinate derivatives were reported from many genus, such as from the lichen *Usnea ceratina* [21], and *Sticta nylanderiana* [22], while orsellinic acid esterified with hydroxylated, short-chain hydrocarbons were found mainly in the genus of *Chaetomium,* if not exclusively. These included globosumones A-C isolated from the endophytic fungus *C. globosum* on Mormon tea [11], which all had a five-carbon side chain, with globosumones A and B displaying moderate cytotoxicity. Seven additional analogs were reported from *C. globosporum* (cib-132) derived from wheat root, incorporating side chains of different sizes, including five-carbon (three compounds), four-carbon (two compounds), and eight-carbon (two compounds) [12]. Elatumone A and B with five-, and eight-carbon side chains, respectively, were isolated from *C. elatum* derived from *Isaria cicadae* [13]. In this study, orsellinic acid esterified with a five-carbon chain (**6** and **7**), four-carbon chain (**9**), and eight-carbon chain (**10**) were also identified, together with a two-carbon chain fused compound (**8**), and orsellinic acid methyl ester (**11**).

In particular, orsellinic acid ester glycosides, orsellides A–E, with antibacterial activities, were isolated from *Chaetomium* sp. (strain Gö 100/9) [10], an endophyte from marine algae, together with the re-isolation of globosumones A and B. The ^13^C-labelled experiments revealed that the sugar moieties of orsellides A–E and the side chains of globosumones A and B all originated from carbohydrate intermediates [10]. In this study, two new orsellinic acid ester glycosides, chaetorsellides A (**1**) and B (**2**) were isolated, together with the known glycoside orsellides A–C. Our study represents the second finding of ester glycosides of orsellinic acid from fungi by checking the Scifinder database (accessed date: 16 August 2026), though phenolic *O*-glycoside of orsellinic acid (such as its 2-*O*-*β*-D-glucopyranoside [23]) has been documented. Moreover, the newly found ester glycosides differed significantly from the known ones, in the esterification site (C-3′ vs. C-2′/C-4′), and the 6-deoxyhexose structure (C-1′: oxymethylene vs. methylated acetal or olefin). Since these two types were co-isolated in the titled fungus SV7, they should be correlated regarding their biosynthesis. Following the initial proposed biosynthetic pathway for orsellides A–E [10], we put forth the hypothetic pathway for the formation of **1** and **2** (Scheme 1A). Starting from D-glucose, consecutive deoxygenations should lead to **1a**, which could be esterified with orsellinic acid at C-3′ to give compound **1**; Meanwhile, dehydrogenation of **1a** should give rise to intermediate **1b** containing a 3′-keto group. Ketalization between **1b** and orsellinic acid could yield the semi-ketal **1c**, which was dehydrated to give **2**. Alternatively, a further dehydrogenation of **1** could yield **2** directly.

Since the five-carbon side chain of globosumones A and B was proved to be of carbohydrate origin (from 5-deoxypentose) [10], it is likely that the other five-carbon analogs should have a similar biosynthesis. The biosynthetic steps for the other short-chain esters are still unknown. It could be possible that the four-carbon type would have a corresponding carbohydrate intermediate, which might derivatize from a pentose via decarboxylation. The eight-carbon derivatives instead might be formed via addition of a C4 unit (such as malic acid) to a pent-carbon side chain, such as the proposed pathway for the formation of elatumone B (**10**) (Scheme 1B). These hypothesis should be tested using isotope-labeling substrates in future.

Orsellinic acid esters have been reported to display a broad spectrum of bioactivities, including antibacterial activities [24], but only a few studies have reported their antifungal activity, and the existing evidence indicates that orsellinic acid exhibits an inhibitory effect against *Aspergillus niger* [25]. However, no antifungal activities have been reported against the phytopathogenic fungi to our knowledge.

In this study, all the isolated orsellinates have been evaluated for their inhibition against the plant infection by *M. oryzae*. All compounds except **4** and **5**, displayed inhibition against the rice blast pathogen infection at 100 μg/mL (Table 4), with **8** and **3** being the most pronounced. These compounds have the potential to be developed as natural fungicides, providing one additional eco-friendly option for sustainable disease management towards rice blast disease. Though **4** and **5** appeared as an inseparable mixture due to their close chemical properties, it seems that this has no obvious effect on the bioassay since a negative result was obtained using the mixture.

It is not possible to draw a clear structure–activity relationship from these data (Table 4). However, some trends could be seen. Regarding the glycoside-type (**1**–**5**), 4′-hydroxylation or glycosidation negatively affects the potency, as **1**–**3** all without a 4′-substitutent displayed inhibition in the range of 20.6–80.5%, while **4** and **5** were inactive, when tested at 100 μg/mL. In addition, 1′-methoxylation might increase the bioactivity when compared to the inhibitory rate of **1** and **2**, vs. **3**. These indicate a suitable polarity is required for the antifungal activity of these compounds. When it comes to compounds **6**–**11**, the polarity rule is still applicable. As could be seen, compound **9** had a free carboxylic group and hydroxyl group and was the least active among these six compounds, while **8** and **11** with less polarity were more potent. Meanwhile, **8** has a di-ester functionality, and is apparently more polar than **11**, but showing the strongest inhibition. Hence, esterification of orsellinic acid with fragments of suitable polarity is important in order to exert its antifungal activity. This insight might guide future structural modifications on orsellinates to maximize their antifungal potency.

As mentioned previously, the antifungal activity of orsellinic acid derivatives has been less investigated, nor is its antifungal mechanism known. As for the isolated compounds in this study, the mechanism of action is still under investigation.

In addition to their direct antifungal activity, some orsellinic acid derivatives like 3,5-dimethyl-orsellinic acid have been reported to activate host (soybean) immune responses and enhance plant disease resistance [26]. These compounds also possess antioxidant activity [27], which can alleviate oxidative damage triggered by pathogen infection. It is likely that the isolated orsellinic acid esters might have similar roles in protecting the host plant, though not yet tested in the rice-*M. oryzae* pathosystem, which requires extensive study in the future. Moreover, from the aspect of endophyte–plant interaction, the interaction of the endophyte SV7 and its metabolites to the host plant foxtail, and whether the *in planta* production of these metabolites confers resistance to plant disease remains to be investigated.

Plant–microbe interactions have a broad scope of context, including their defense–antidefense, mutual benefit, adaption, and co-evolution, etc. Understanding these interactions are of utmost interest, not only enriching the knowledge of their mechanism of interactions, but also contributing to crop protection. Our study provides one more example for exploring bioactive (antifungal) substances from plant–endophyte interaction.

## 4. Materials and Methods

### 4.1. General Experimental Procedures

Sephadex LH-20 (Amersham Pharmacia Biotech, Uppsala, Sweden), and ODS gel (octadecylsilane-modified silica gel) (YMC CO., LTD., Kyoto, Japan) were employed as materials for chromatographic isolation. HPLC-DAD analysis was performed on a Shimadzu LC-20A instrument equipping with a SPD-M20A photodiode array detector (Shimadzu Corp., Tokyo, Japan) using an analytic C18 column (250 mm × 4.6 mm i.d., 5 μm; Phenomenex Inc., Torrance, CA, USA). Semi-HPLC separation was accomplished with a Lumtech chromatographic system (Lumiere Tech. Ltd., Beijing, China). The system was configured with a K-501 pump set to a flow rate of 3 mL/min and a K-2501 UV detector (detection wavelength 210 nm), in conjunction with a Luna-C_18_ column (250 mm × 10 mm i.d., 5 μm, Phenomenex Inc., Torrance, CA, USA).

Optical rotations were recorded on a Rudolph Autopol III automatic polarimeter (Rudolph Research Analytical, Hackettstown, NJ, USA). Circular dichroism (CD) spectra were recorded on a JASCO J-815 CD spectrometer (JASCO Corp., Tokyo, Japan). High-resolution electrospray ionization mass spectrometry (HRESIMS) spectra were recorded on a LC 1260/Q-TOF-MS 6520 machine (Agilent Technologies, Santa Clara, CA, USA). ^1^H, ^13^C, and 2D NMR spectra were measured on a Bruker Avance 500 NMR spectrometer (Bruker BioSpin, Zürich, Switzerland). NMR chemical shifts were given in δ (ppm), referring to the residual solvent peaks of CD_3_OD at δ_H_ 3.31 and δ_C_ 49.0, and coupling constants in Hertz.

### 4.2. Fungal Material

The endophytic fungi SV7 was isolated from the inner tissue of green foxtail *S*. *viridis* collected from the experimental field on the campus of China Agricultural University (CAU). It was identified as *C*. *novozelandicum* based on morphological observation and molecular biological identification. Its ITS rDNA sequence (GenBank accession PZ798797) shared 99.61% sequence identity with *C*. *novozelandicum* CBS 124555 (GenBank accession: NR_144857.1) (accessed date: 16 August 2026).

### 4.3. Fermentation, Extraction, and Isolation

#### 4.3.1. Small-Scale Fermentation

The isolated endophytic fungi from *S. viridis* were fermented on rice medium (rice 30 g, deionized water 33 mL), which was chosen due to its ability to induce abundant and reproducible fungal secondary metabolites [4,5], under static conditions, at 25 °C in the dark for 15 days, before harvest and extraction with EtOAc. The EtOAc extracts were subjected to mycelial growth rate assay (see Section 4.4.1), and HPLC-DAD analysis.

#### 4.3.2. Large-Scale Fermentation of SV7, Chemical Extraction, and Isolation

A seed culture of fungal strain SV7 was prepared in liquid PDB medium with mycelia plugs sliced from agar plate, and kept in dark at 25 °C, 180 rpm for 7 days. The seed culture was transferred into sterilized rice medium in a 1 L flask (each contained rice 100 g, deionized water 110 mL), and a total of 50 flasks were used, fermented for 30 days before harvest. The culture was extracted with EtOAc three times, which was concentrated using rotary evaporator under vacuum to give the crude organic extract. This was then defatted with petroleum ether to afford 27.5 g of crude extract.

The crude extract was dissolved in methanol and divided into ten equal portions, each of which was subjected to column chromatography over Sephadex LH-20, by eluting with a mixture of methanol and dichloromethane (1:1, *v*/*v*) using the identical procedure. The fractions were pooled, resulting in a total of thirteen fractions (Fr. 1–13).

Among these, Fr. 13 was purified using semi-preparative HPLC eluting with 22% Acetonitrile/H_2_O to give compound **9** (2.5 mg). Fr. 7–12 were combined due to their similar HPLC profiles, which were chromatographed over ODS, eluting with a gradient of methanol in water from 25% to 80%. A total of 24 subfractions (Fr. B1–B24) were collected. From subfraction Fr. B13, five compounds were isolated, by using semi-preparative HPLC with 25% acetonitrile in water as the mobile phase, including compound **2** (4.7 mg), **6** (2.6 mg), **7** (3.3 mg), and **10** (5.2 mg). Similarly, compound **1** (2.4 mg), **4** and **5** (14.5 mg, an inseparable mixture with ratio of 4/3 as determined by ^1^H NMR), **11** (1.8 mg), and **8** (2.7 mg) were purified from subfraction Fr. B16 by semi-preparative HPLC eluting with 32% acetonitrile/H_2_O. Likewise, compound **3** (1.5 mg) was isolated from subfraction Fr. B19 using 45% acetonitrile/H_2_O as the mobile phase.

**Chaetorsellide A** (**1**): pale-yellow amorphous powder; [α]^27^_D_ +60 (*c* 0.1, MeOH); UV (MeOH) λmax 199, 215, 263, 298 nm; ^1^H NMR (CD_3_OD, 500 MHz), ^13^C NMR (CD_3_OD, 125 MHz) see Table 1. HRESIMS *m*/*z* 281.1012 [M − H]^−^ (calcd for C_14_H_17_O_6_^−^, 281.1031).

**Chaetorsellide B** (**2**): pale-yellow amorphous powder; [α]^27^_D_ +15 (*c* 0.2, MeOH); UV (MeOH) λmax 198, 214, 226, 268, 300 (sh) nm; ECD (0.27 mg/mL, MeOH) λ (mdeg) 215 (+11.00), 228 (+10.44), 253 (−1.83), 271 (+0.82), 301 (−2.53) nm; ^1^H NMR (CD_3_OD, 500 MHz), ^13^C NMR (CD_3_OD, 125 MHz) see Table 1. HRESIMS *m*/*z* 279.0885 [M − H]^−^ (calcd for C_14_H_15_O_6_^−^, 279.0874).

**Chaetorsellide C** (**6**): pale-yellow amorphous powder; [α]^26^_D_ -20 (*c* 0.3, MeOH); UV (MeOH) λmax 198, 216, 262, 299 nm. ^1^H NMR (CD_3_OD, 500 MHz), ^13^C NMR (CD_3_OD, 125 MHz) see Table 3. HRESIMS *m*/*z* 269.1016 [M − H]^−^ (calcd for C_13_H_17_O_6_^−^, 269.1031).

**Chaetorsellide D** (**8**): pale-yellow amorphous powder, UV (MeOH) λmax 198, 216, 264, 298 nm. ^1^H NMR (CD_3_OD, 500 MHz), ^13^C NMR (CD_3_OD, 125 MHz) see Table 3. HRESIMS *m*/*z* 263.0537 [M + Na]^+^ (calcd for C_11_H_12_O_6_Na^+^, 263.0526).

### 4.4. Antifungal Assay Against the Rice Blast Pathogen

#### 4.4.1. Mycelial Growth Rate Assay

The crude extract of each fungal culture was evaluated against the rice blast pathogen *M. oryzae* using the mycelial growth rate assay as described previously [28]. Briefly, the crude extract was dissolved in DMSO, and added to oatmeal tomato agar (OTA) medium at a given concentration. In this study, a final concentration of 100 μg/mL was employed. Then, mycelial disk (5 mm in diameter) of *M. oryzae* P131 was inoculated onto the middle of a OTA plate at 28 °C for 7 days. The radial lengths of the mycelia were compared with DMSO which served as a negative control. Carbendazim was used as a positive control. Three replicates for each sample concentration were tested.

#### 4.4.2. Confrontation Assay

*M*. *oryzae* was inoculated 2 cm away from the center of a 9 cm PDA plate, while SV7 was inoculated on the opposite side along the same diameter line, also 2 cm from the center. Three biological replicates were performed, and plates inoculated only with the pathogen were set as controls. Inoculated plates were incubated at 25 °C and examined daily for 5–7 days.

Inhibition rate (%) = [(radial growth of control − radial growth of treatment)/radial growth of control] × 100%

#### 4.4.3. Plant Infection Assay

The plant infection assays were performed on seedling leaves of barley or rice leaves, as described previously [28,29].

Briefly, *M. oryzae* P131 was grown on OTA plates at 28 °C to induce conidia. For initial assay, six-day-old barley seedlings were used. The isolated compounds were adjusted to concentrations of 10 mg/mL and 2.5 mg/mL in DMSO as stock solution. The stock solution was thoroughly mixed with *M. oryzae* conidial suspension (10^5^ conidia/mL) at a volume ratio of 1:100, and the mixture was drop-inoculated onto barley leaves. Each experiment was repeated six times independently. Infected plants were incubated in a humidity chamber at 28 °C. Disease lesions on barley leaves were examined at 5 days post-inoculation (dpi).

For rice leaf inoculation, stock solutions were prepared at 10 mg/mL, 5 mg/mL and 2.5 mg/mL, respectively, and the resulting mixtures were wound-inoculated onto rice leaves. Seedlings of susceptible rice cultivar ‘NPB’ at the four-leaf stage were used. Each experiment was repeated nine times independently.

Inhibition rate (%) = (Lesion area of control group − Lesion area of experimental group)/Lesion area of control group × 100%

### 4.5. Statistical Analysis

Lesion areas (cm^2^) for each test group were measured using Image J 2026 1.54t software. Mean values were calculated from independent experiments (*n* = 6 for barely leaves, *n* = 9 for rice leaves), and the results were expressed as mean ± SD. GraphPad Prism 9.5.1 software was used for data processing and significance analysis by one-way analysis of variance (ANOVA).

### 4.6. Quantum-Chemical Calculation for Compound **2**

#### 4.6.1. ECD Calculation Method

Conformational searches were carried out using Merck Molecular Force Field (MMFF). Geometry optimization using the DFT method was performed at the B3LYP/6-31+G(d,p) level by Gaussian 09, as described previously [30]. The equilibrium population of each conformer at 298.15 K was calculated from its relative Gibbs free energies using Boltzmann statistics. TDDFT ECD calculations of the low-energy conformers (≥1%) without imaginary frequencies were performed with Gaussian 09 using PBE0 functional and TZVP basis set with the polarizable continuum model (PCM) for MeOH. The ECD spectrum of each conformer was simulated by the program SpecDis [31] using a Gaussian band shape with an exponential half-width σ of 0.2 eV, using dipole-length computed rotational strengths. The Boltzmann-averaged ECD spectrum was generated according to the Boltzmann distributions of the lowest energy conformers for each structure. Theoretical ECD spectra were then compared with the experimental one to determine the absolute configuration. The calculated ECD spectra were UV-shifted (+10 nm), for comparison with the measured spectrum.

#### 4.6.2. ^13^C NMR Calculation

The ^13^C NMR calculations were performed at the level of mPW1PW91/6-311+G(2d,p) using the gauge-independent atomic orbital (GIAO) method, with the solvent effect included (PCM = methanol). The chemical shift for each atom was considered as the average value of the same atom in different conformers according to their Boltzmann distributions, using the relative Gibbs free energies as weighting factors. The empirical scaling method was used to decrease the systematic error. The computed isotropic shielding constant (σ) for each atom was converted to the scaled chemical shift value (δcal, relative to TMS) using the following equation, *δ*cal = (186.5993 − σ)/1.0399, in which the scaling factors (the slope and intercept) were obtained from the linear regression analyses of experimental and computed chemical shift data for known compounds, as reported by Pierens et al. [32]. The results are summarized in Table 2. The calculated values (*δ*cal) were then compared with the experimental ones (*δ*exp), and their differences were evaluated in terms of the average absolute deviation, maximum absolute deviation, and the RMSD value.

## 5. Conclusions

In this study, the endophytic fungi were isolated from green foxtail *S. viridis*, and screened for their activity against the rice blast pathogen. Among them, the extract of *C. novozelandicum* SV7 showed the best inhibition using the mycelia growth rate assay. A large-scale fermentation, extraction, and detailed chromatographic separation of this fungus led to the isolation and identification of eleven secondary metabolites of the orsellinate type. Among them, four (**1**, **2**, **6**, and **8**) were new compounds, five (**1**–**5**) were orsellinic acid ester glycosides. This was the first report of secondary metabolite from *C. novozelandicum*. These compounds were evaluated for their inhibition against the rice blast pathogen using the plant infection assay, among which compounds **8** and **3** displayed significant inhibition, with compound **8** being the most powerful one. These metabolites have potential to be developed as natural fungicides.

The mechanism of action of these orsellinic acid esters are as yet unknown. Future work should unveil the molecular target of these compounds using modern molecular techniques, including transcriptomic, metabolomic, and proteomic analysis. Meanwhile, the antifungal spectrum of these metabolites should be determined by testing against a variety of phytopathogenic fungi.

However, the limited amounts of these metabolites hinder further detailed biological investigation, nor was the stability of these compounds studied. Sufficient quantities of minor metabolites could be accumulated by repeated workup on large-scale fermentation. Alternatively, the product yields could be increased, by further optimization of the fermentation, or engineering the biosynthetic pathway which is unknown at present. In addition, the synthetic and/or biosynthetic methods to generate these metabolites are highly demanding. If these were feasible, structural modifications on these scaffolds should lead to optimized structures with higher potency.

Further, the interaction between the endophyte SV7 and the host plant should be examined *in planta*, and whether the metabolites of SV7 can enhance plant resistance to pathogens by activating an immunity response, in addition to their direct antifungal effects, as in the case of 3,5-dimethyl-orsellinic acid [26], is unknown, but the research is intriguing and ongoing in our lab.

Ultimately, such efforts may pave the way toward the practical application of these compounds as promising candidates for environmentally friendly crop protection, and also strengthen the rationale for exploring bioactive substances from whence they are produced (i.e., plant–microbe interaction).

## Figures and Tables

**Figure 1:**
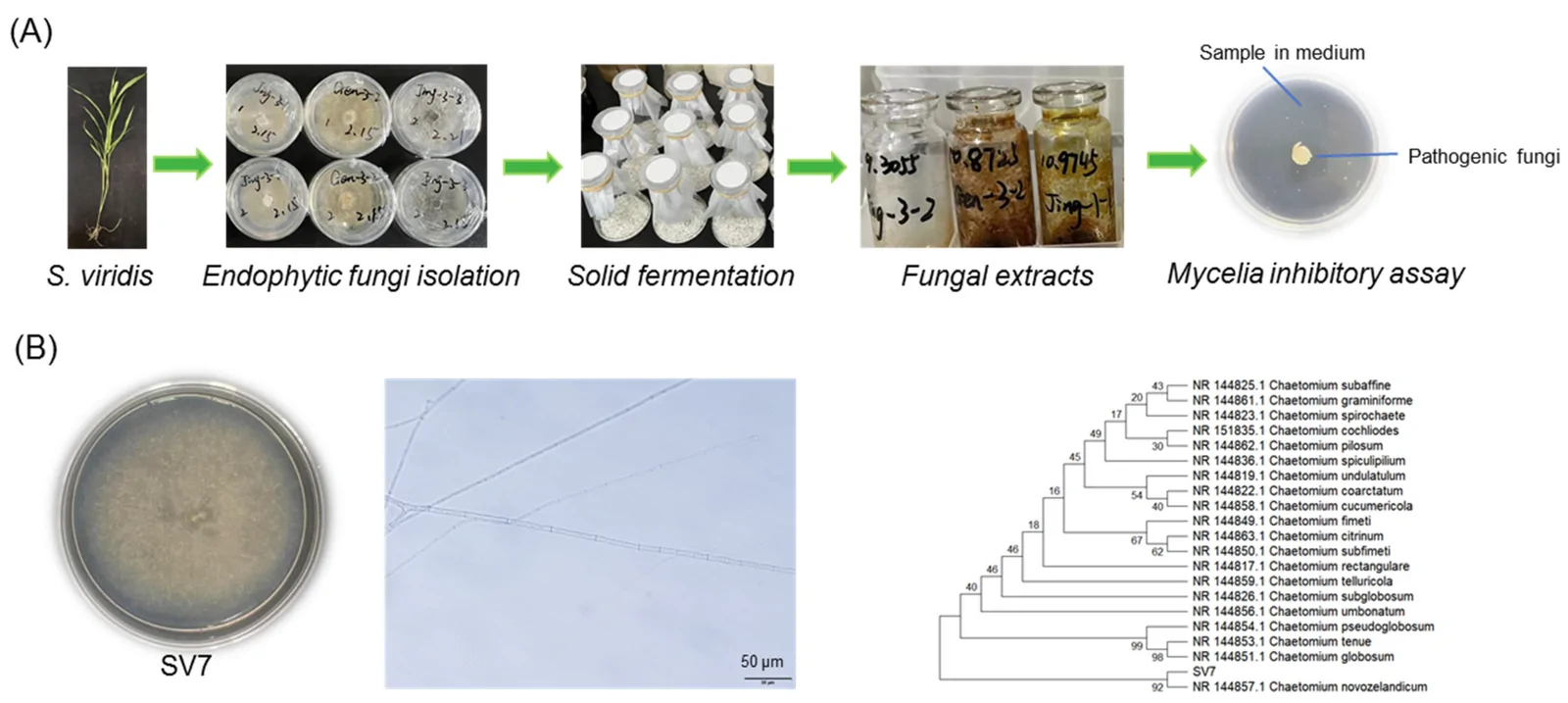
A general workflow to scrutinize the endophytic fungi for anti-phytopathogenic activities (**A**), and the identification of the endophytic fungus SV7 (**B**).

**Figure 2:**
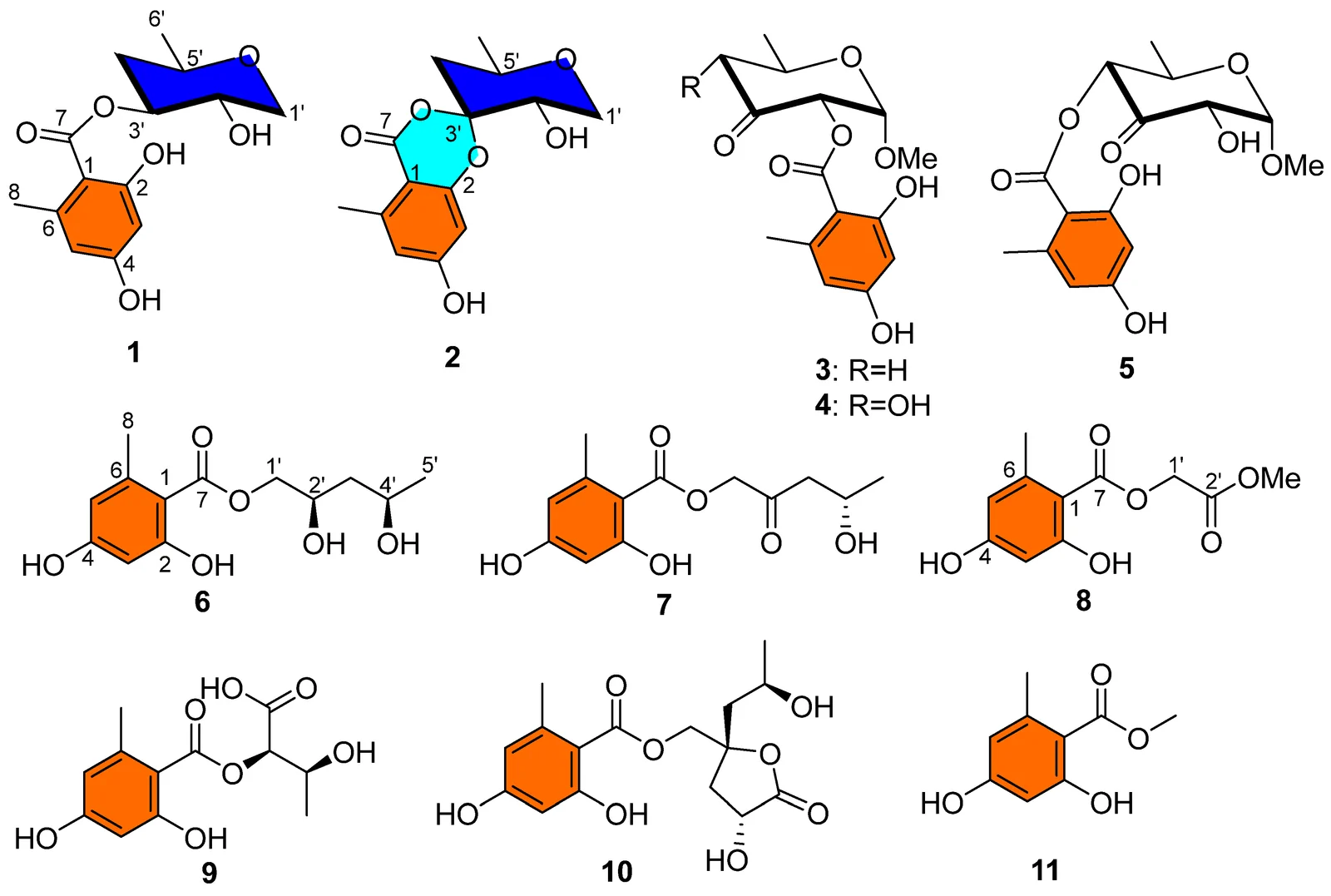
The isolated compounds from the endophytic fungus SV7. Note: compounds **1**–**2**, **6**, and **8** are new compounds.

**Figure 3:**
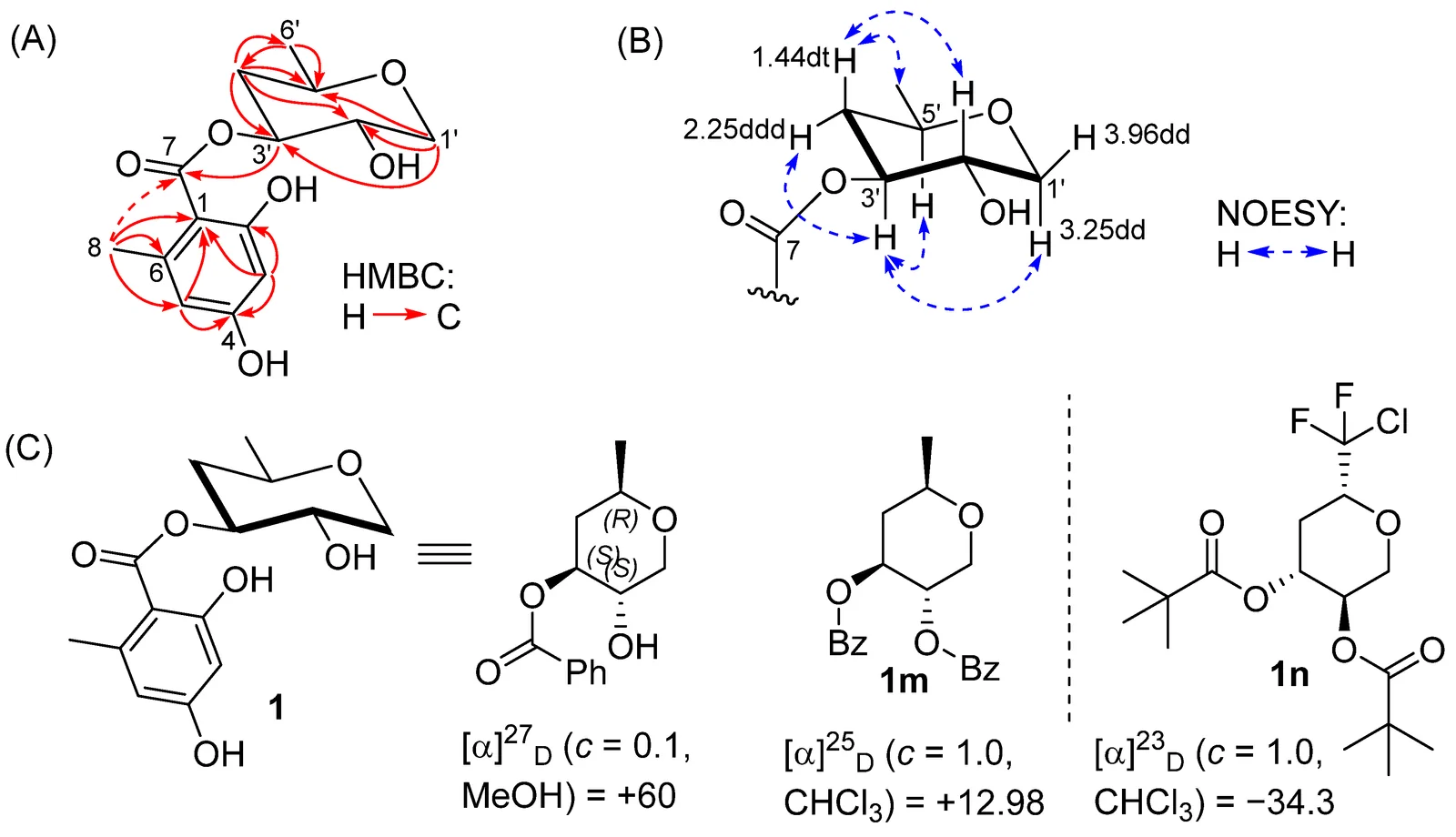
Selected HMBC (**A**), and NOESY (**B**) correlations of **1**, and comparison of the optical rotation with the known analogs (**C**).

**Figure 4:**
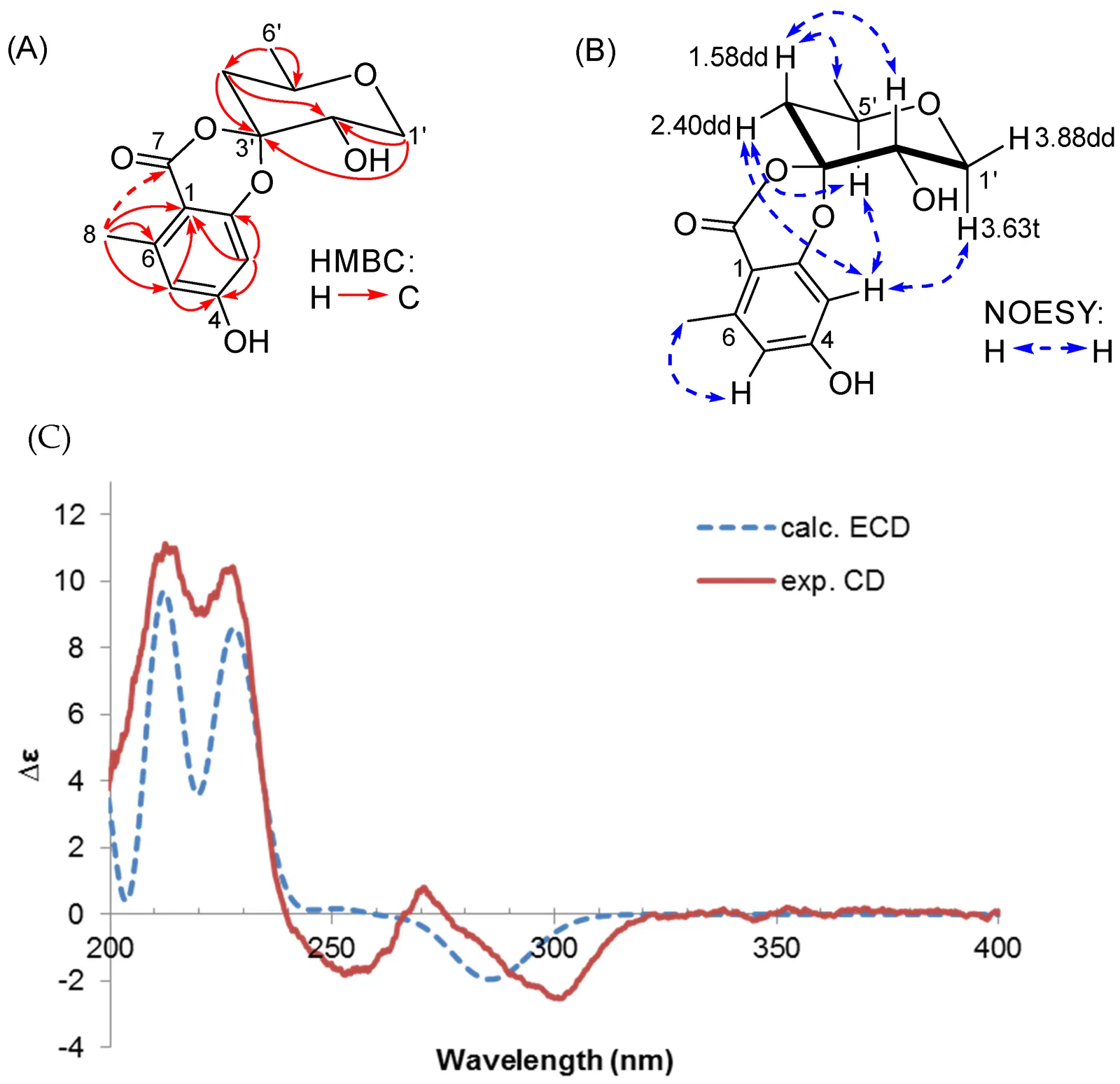
Selected HMBC (**A**), and NOESY (**B**) correlations of **2**, and comparison of the ECD spectrum of **2** with the calculated one (**C**).

**Figure 5:**
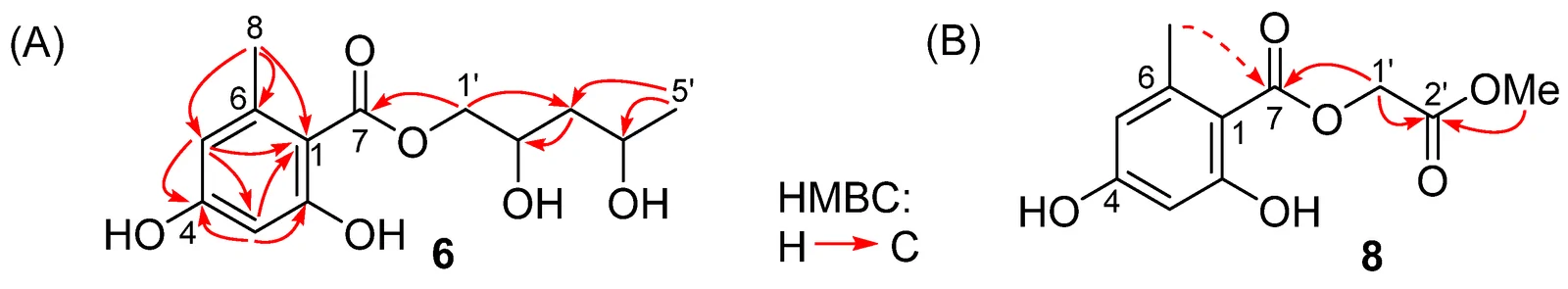
Selected HMBC correlations of **6** (**A**) and **8** (**B**).

**Figure 6:**
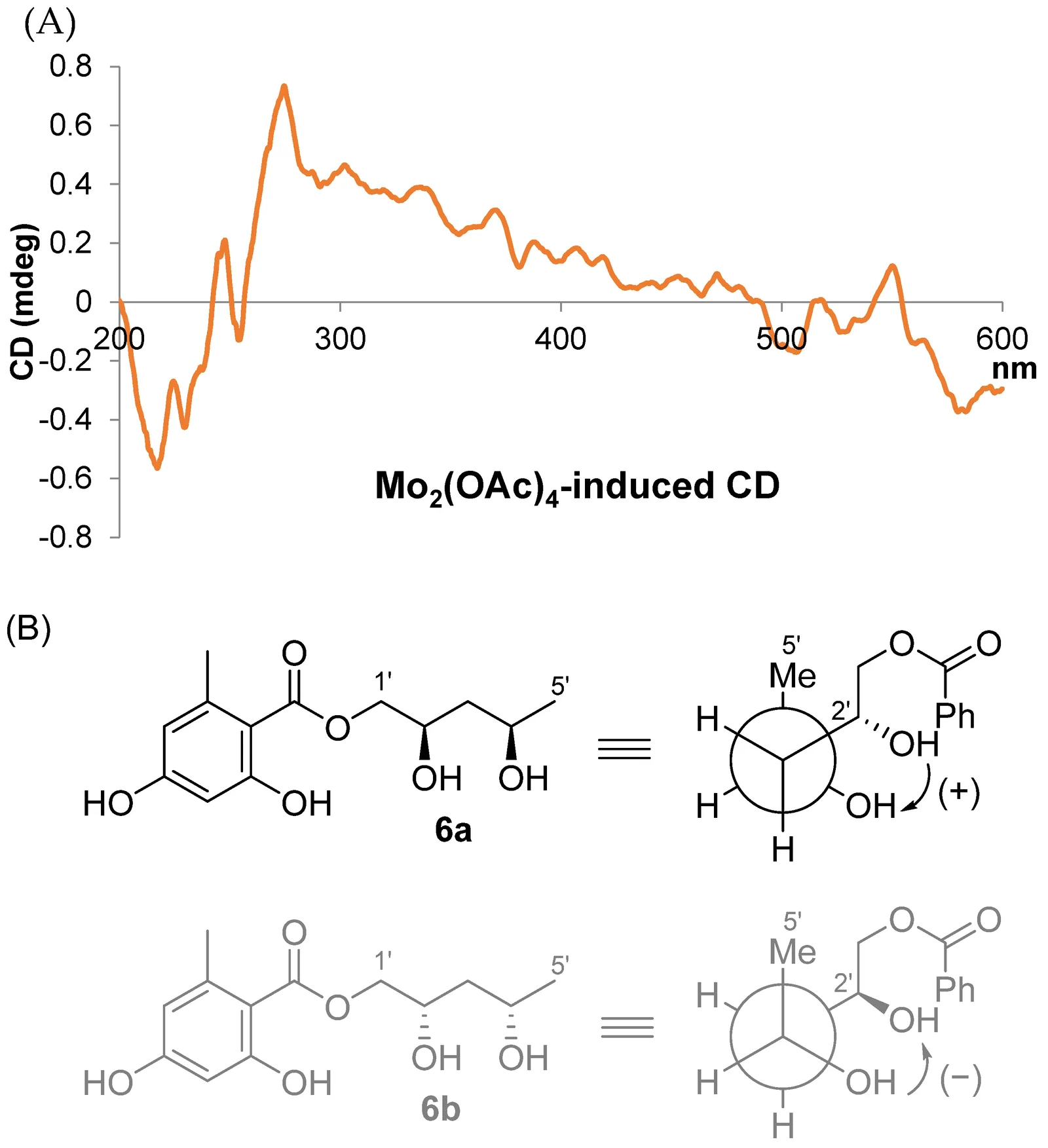
Mo_2_(AcO)_4_-induced CD spectrum of **6** (**A**), indicating a *syn*-relationship between the hydroxyl groups as depicted in **6a** (**B**).

**Figure 7:**
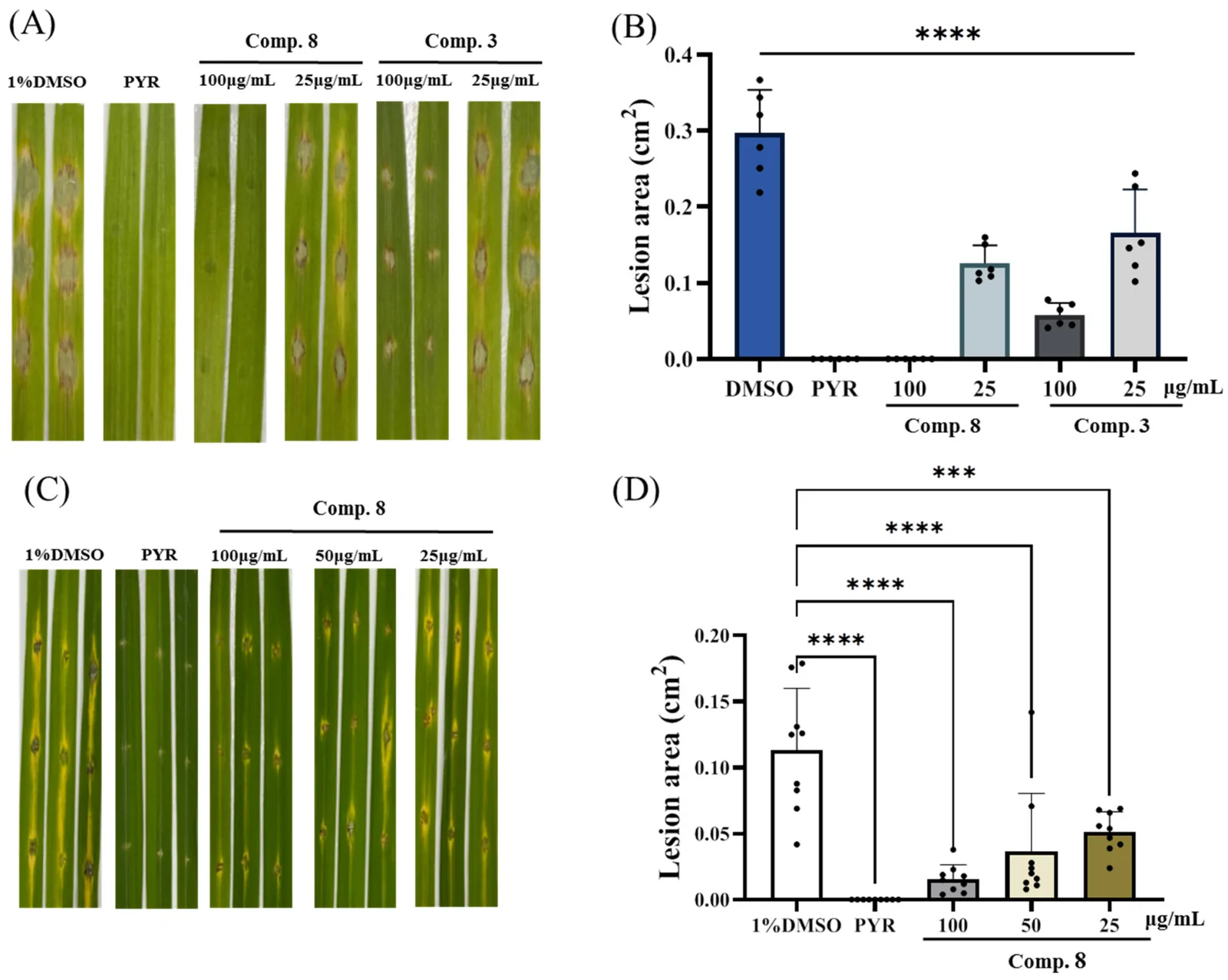
The plant infection assays on barley leaves ((**A**,**B**), drop-inoculated), and on rice leaves ((**C**,**D**), wound-inoculated). Statistical analysis of lesion areas (cm^2^) on barley leaves (**B**) or rice leaves (**D**). Data are presented as mean ± standard deviation and analyzed with one-way analysis of variance (ANOVA) (***, *p* < 0.001; ****, *p* < 0.0001; *n* = 6 in (**B**), *n* = 9 in (**D**)).

**Scheme 1:**
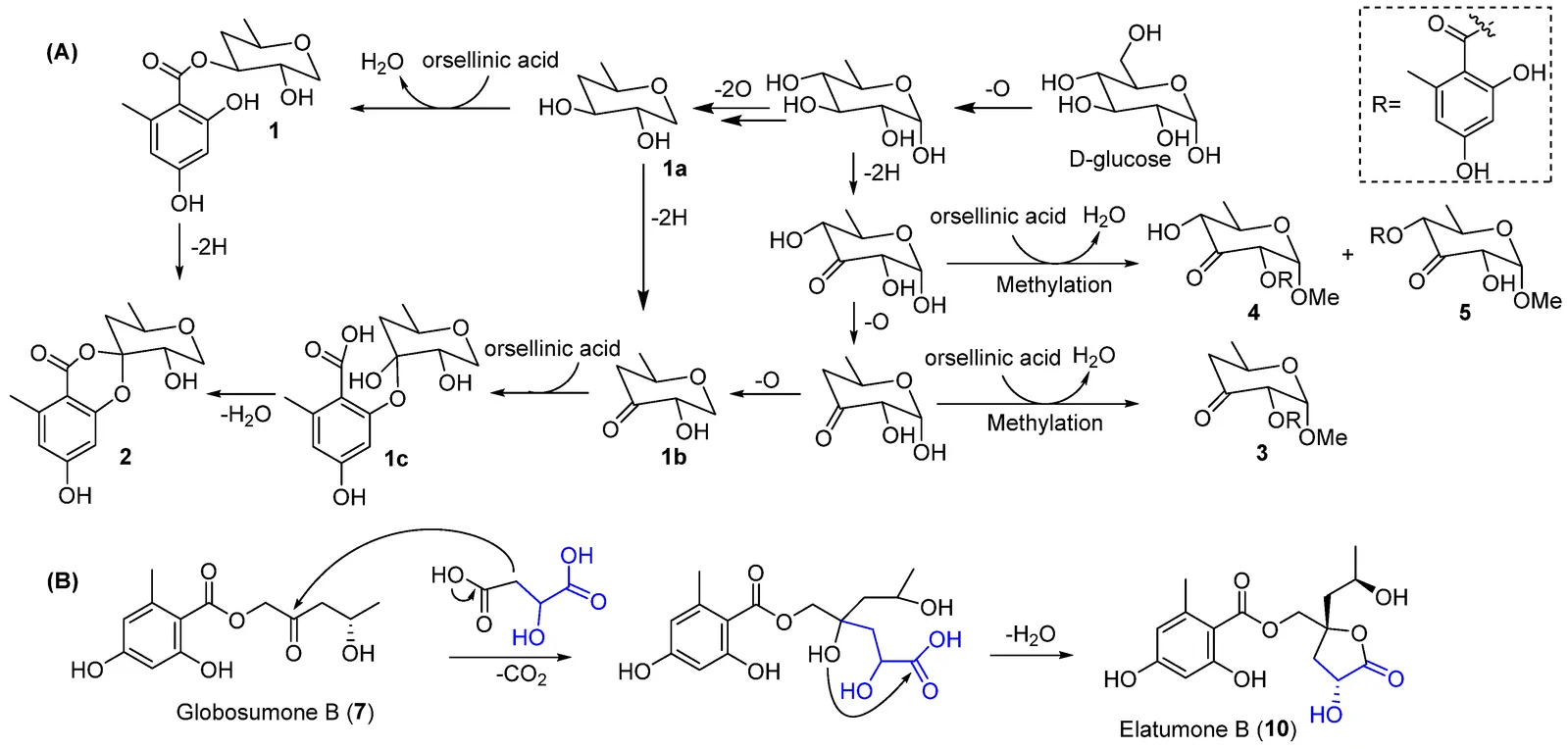
Proposed biosynthetic pathway for the orsellinic acid ester glycosides (**1**–**5**) (**A**), and the eight-carbon side chain fused orsellinic acid ester (**10**) (**B**).

**Table 1:** ^1^H (500 MHz) and ^13^C (125 MHz) NMR data of compounds **1** and **2** (Methanol-*d*_4_).

	1		2	
Position	*δ*_H_ Mult. (*J* in Hz)	*δ*_C_ Type	*δ*_H_, Mult. (*J* in Hz)	*δ*_C_ Type
1		105.9 C		104.95 C
2		166.3 C		160.0 C
3	6.16 d (2.5)	101.7 CH	6.34 d (2.3)	102.3 CH
4		163.9 C		165.6 C
5	6.21 d (2.5)	112.6 CH	6.42 d (2.4)	115.0 CH
6		144.8 C		146.8 C
7		172.6 C		162.0 C
8	2.52 s	24.6 CH_3_	2.55 s	22.3 CH_3_
1′	3.96 dd (11.3,5.5) 3.25 dd (11.3,10.5)	71.3 CH_2_	3.88 dd (10.4,5.5) 3.63 t (10.3)	68.6 CH_2_
2′	3.74 ddd (10.5,9.2,5.5)	69.9 CH	3.84 m	70.9 CH
3′	5.06 ddd (11.3,9.2,5.1)	77.2 CH		105.03 C
4′	2.25 ddd (12.7,5.2,2.0) 1.44 dt (12.7,11.3)	39.5 CH_2_	2.40 dd (14.0,2.1) 1.58 dd (14.0,11.3)	41.3 CH_2_
5′	3.63 dqd (11.3,6.2,2.0)	73.6 CH	3.69 dqd (12.5,6.3,2.0)	71.7 CH
6′	1.21 d (6.2)	21.4 CH_3_	1.11 d (6.3)	20.9 CH_3_

**Table 2:** Calculated ^13^C NMR shift for **2**.

Position	Calculated Chemical Shift (δcal)	Experimental Chemical Shift (δexp)	Difference (δcal-δexp) ^a^
1	104.91	104.95	−0.04
2	159.47	160	−0.53
3	100.35	102.3	−1.95
4	162.73	165.6	−2.87
5	112.12	115	−2.88
6	149.30	146.8	2.50
7	160.26	162	−1.74
8	24.11	22.3	1.81
1′	66.96	68.6	−1.64
2′	71.10	70.9	0.20
3′	105.51	105.03	0.48
4′	39.78	41.3	−1.52
5′	70.76	71.7	−0.94
6′	20.05	20.9	−0.85

^a^ Note: the average absolute difference between the calculated and experimental data is 1.43 ppm, while the RMSD between them is 1.69 ppm.

**Table 3:** ^1^H (500 MHz) and ^13^C (125 MHz) NMR data of compounds **6** and **8** (Methanol-*d*_4_).

	6		8	
Position	*δ*_H_ Mult. (*J* in Hz)	*δ*_C_ Type	*δ*_H_ Mult. (*J* in Hz)	*δ*_C_ Type
1		105.9 C		105.3 C
2		166.2 C		166.2 C
3	6.16 d (2.5)	101.7 CH	6.17 d (2.4)	101.7 CH
4		163.8 C		164.3 C
5	6.21 d (2.5)	112.5 CH	6.24 d (2.4)	112.7 CH
6		144.8 C		145.0 C
7		172.8 C		171.9 C
8	2.51 s	24.5 CH_3_	2.51 s	24.2 CH_3_
1′	4.35 dd (11.4,3.9) 4.27 dd (11.4,6.2)	69.7 CH_2_	4.91 s	61.7 CH_2_
2′	4.10 m	68.9 CH		170.0 C
2′-OMe			3.79 s	52.8 CH_3_
3′	1.73 dt (13.9,8.0) 1.66 dt (13.9,5.1)	43.4 CH_2_		
4′	4.00 m	66.7 CH		
5′	1.21 d (6.2)	23.6 CH_3_		

**Table 4:** Inhibitory rate of the isolated compounds against the plant infection.

Concentration	Inhibitory Rate											
	DMSO	PYR	1	2	3	4 + 5	6	7	8	9	10	11
100 μg/mL	0.0%	100.0%	23.2%	20.6%	80.5%	−5.8%	18.3%	19.2%	100.0%	4.7%	18.5%	30.8%
25 μg/mL	0.0%	100.0%	25.0%	−5.4%	44.1%	−11.2%	11.1%	8.5%	57.6%	−5.2%	6.6%	10.1%

## Data Availability

The data supporting this article have been included in the Supporting Information.

## Supplementary Materials

The following supporting information can be downloaded at: https://www.mdpi.com/article/10.3390/ijms27198582/s1.
