# Supplementary material for: Green Foxtail-Derived Endophytic Fungus Chaetomium novozelandicum SV7 Produced Orsellinic Acid Ester Glycosides Active Against Rice Blast Pathogen
Source: Int J Mol Sci. 2026 Sep 25;27(19):8582. doi: 10.3390/ijms27198582 (PMC13665201; doi:10.3390/ijms27198582)
Supplement: Supplementary file 1 [file ijms-27-08582-s001.zip › ijms-4542455-supplementary.pdf]

Supporting Information

Green foxtail-derived endophytic fungus *Chaetomium novozelandicum* SV7 produced orsellinic acid ester glycosides active against rice blast pathogen

Jianing Wang[1,2,†], Yonglin Yang[2,†], Jinlan He[1,2], Yanxue Peng[2], Yifei Qin[2], Xuan Zhang[2], Zhen Shen[2], Jun Yang[1,2], Ligang Zhou[2], Daowan Lai[1,2,*]

[1] Sanya Institute, China Agricultural University, Sanya 572025, China

[2] State Key Laboratory of Agricultural and Forestry Biosecurity, MARA Key Lab of Pest Monitoring and Green Management, College of Plant Protection, China Agricultural University, Beijing 100193, China

† These authors contributed equally to this work

* Correspondence: dwlai@cau.edu.cn

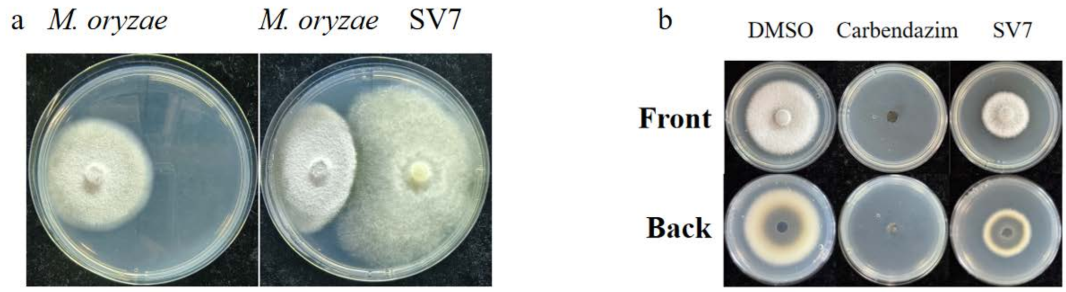

Figure S1. Antifungal Activity Assay of SV7. a: Dual-culture assay of strain SV7 against *M. oryzae*. b: Inhibition assay of SV7 crude extract against *M. oryzae* at 100 μg/mL.

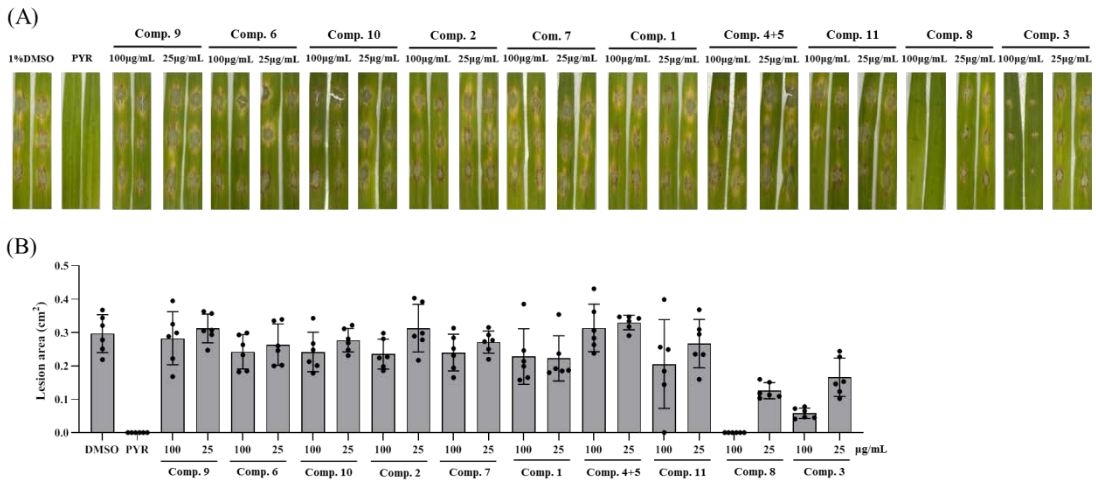

Figure S2. Plant infection assay against the rice blast pathogen on barley leaves for all the isolated compounds

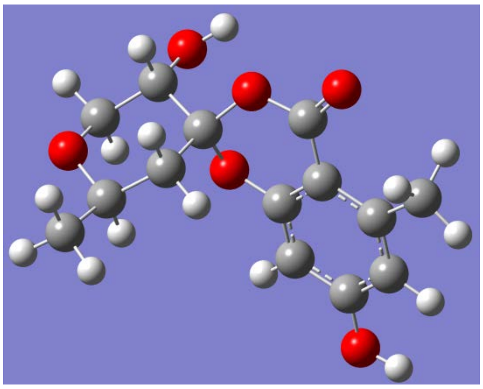

2-11, 31.9%

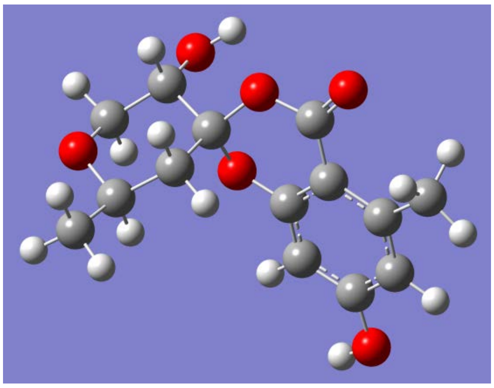

2-12, 28.7%

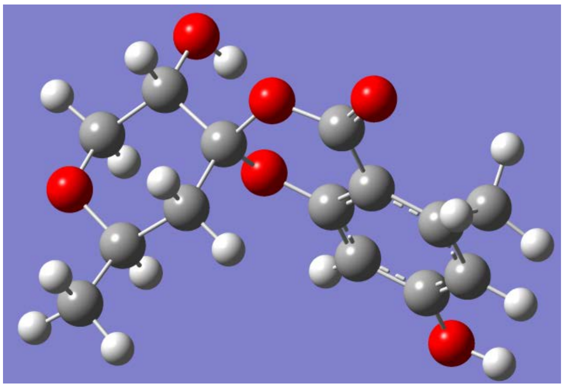

2-5, 17.9%

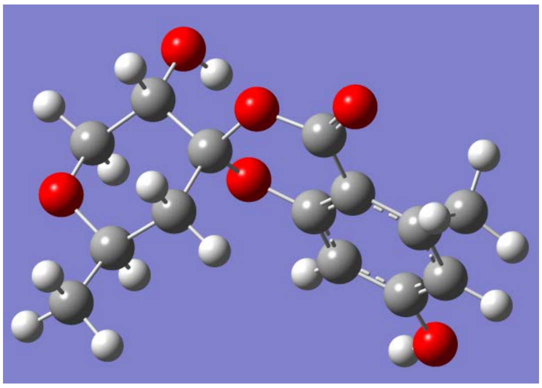

2-7, 11.9%

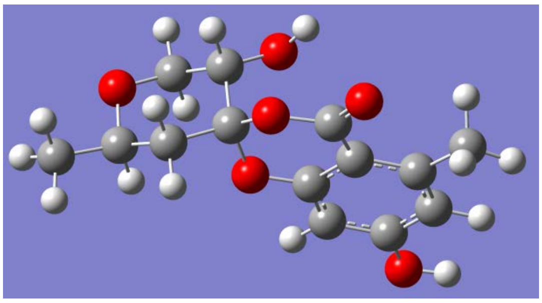

2-8, 3.6%

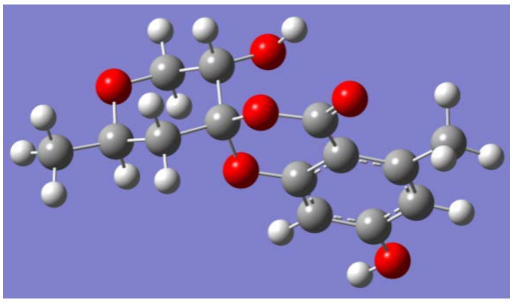

2-9, 3.2%

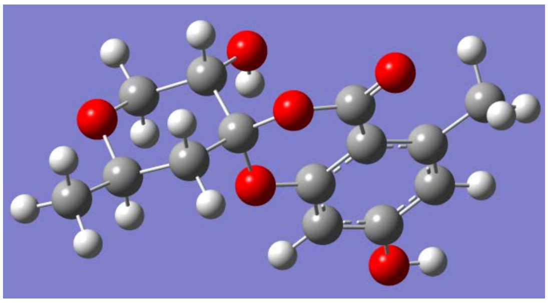

2-6, 1.8%

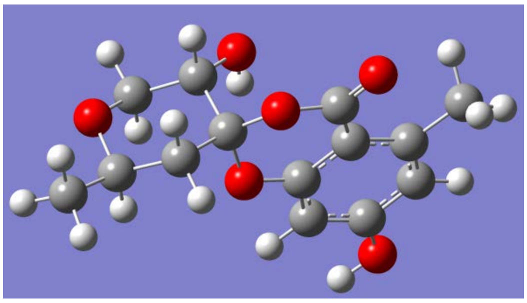

2-10, 1.0%

Figure S3. The major conformers of **2** optimized at B3LYP/6-31+G(d, p) level with their population calculated from its relative Gibbs-free energies.

NMR spectra

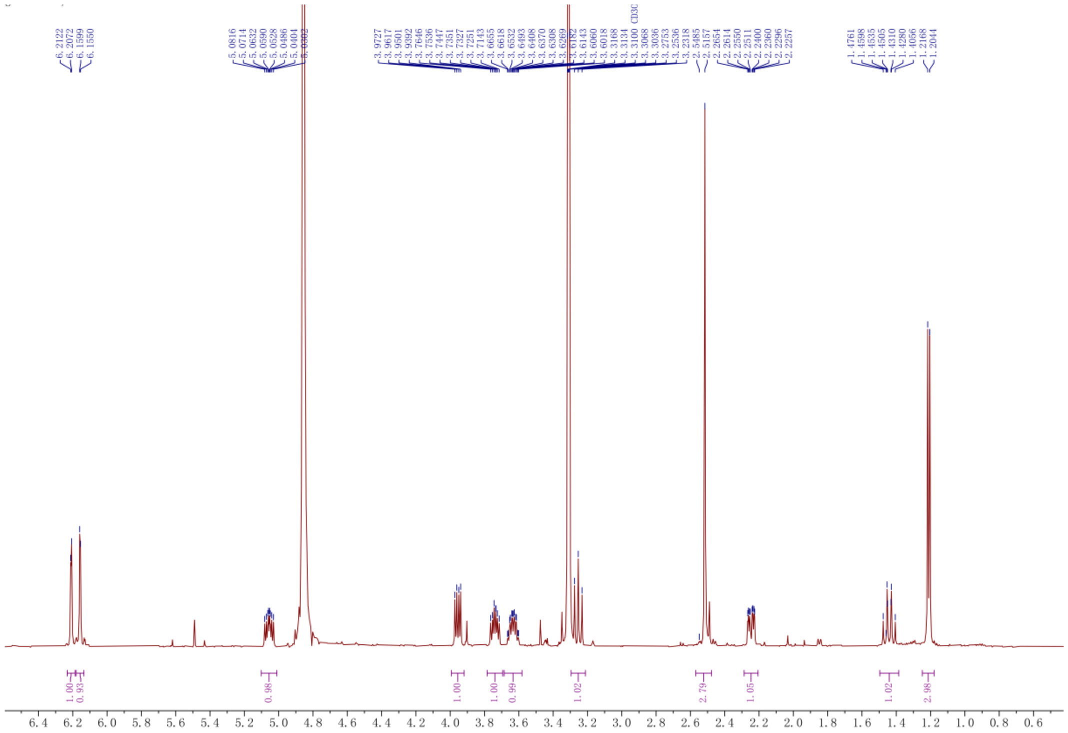

Figure S4-1. ^{1}H NMR spectrum of compound **1** (CD_3OD, 500 MHz)

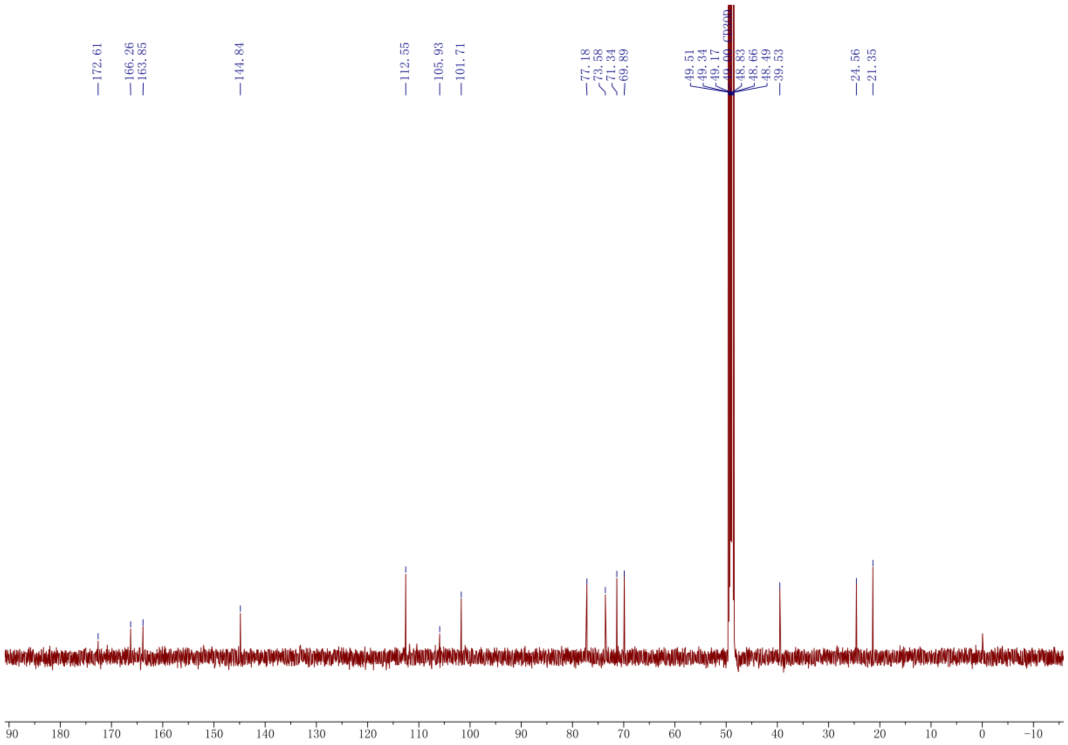

Figure S4-2. ^{13}C NMR spectrum of compound **1** (CD_3OD, 125 MHz)

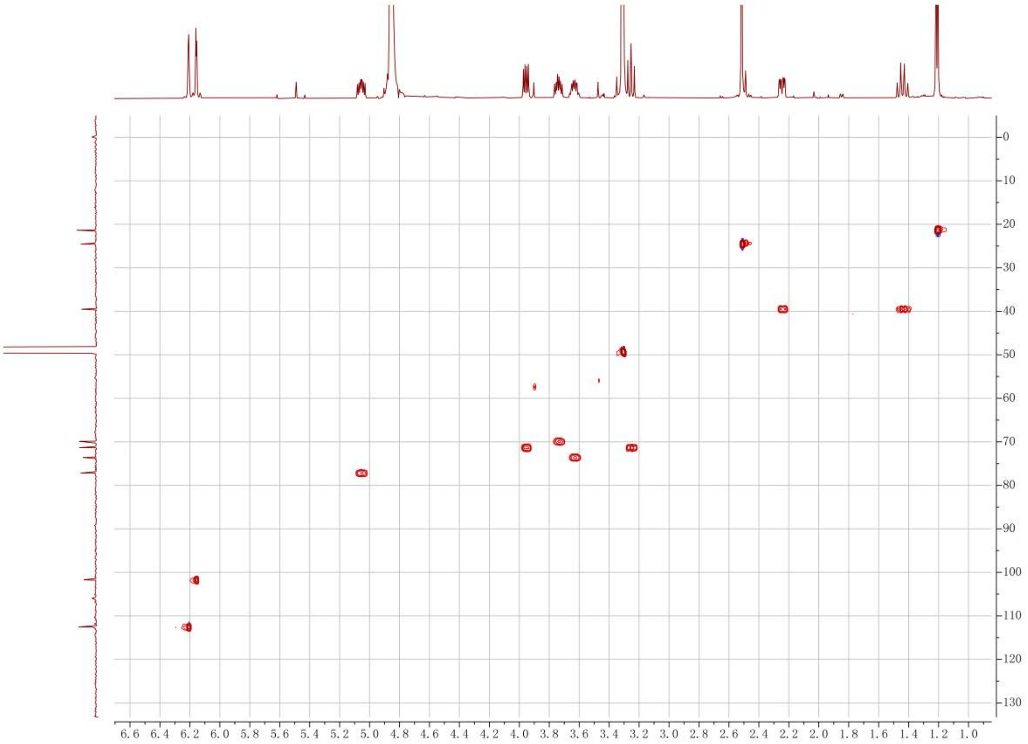

Figure S4-3. HSQC spectrum of compound **1**

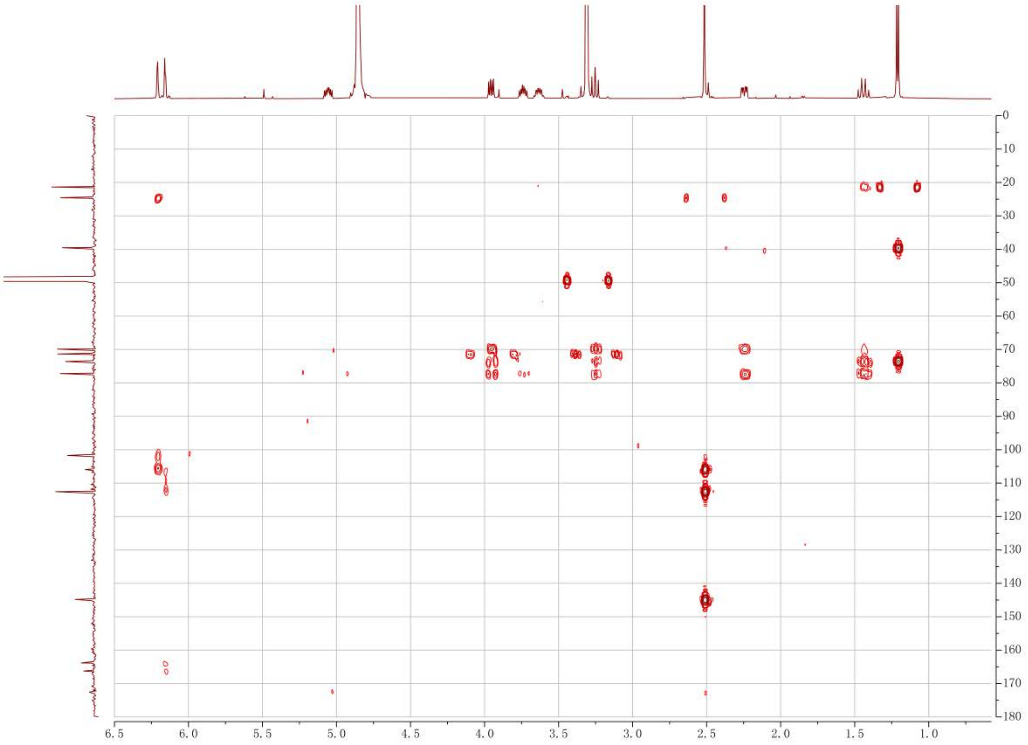

Figure S4-4. HMBC spectrum of compound **1**

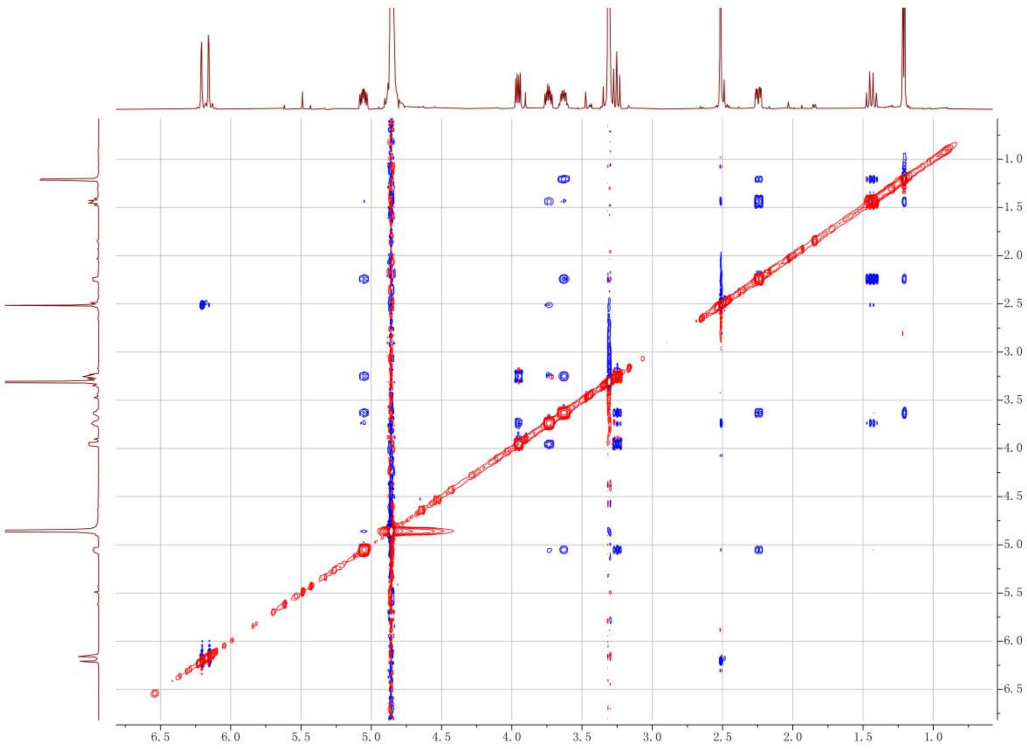

Figure S4-5. NOESY spectrum of compound **1**

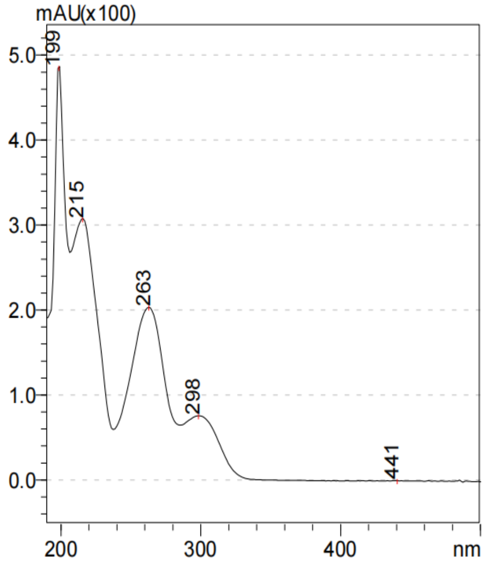

Figure S4-6. UV spectrum of compound **1**

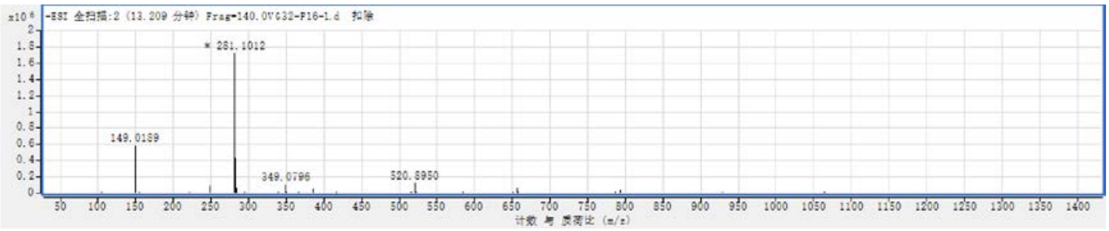

Figure S4-7. HR-ESI-MS spectrum of compound **1**

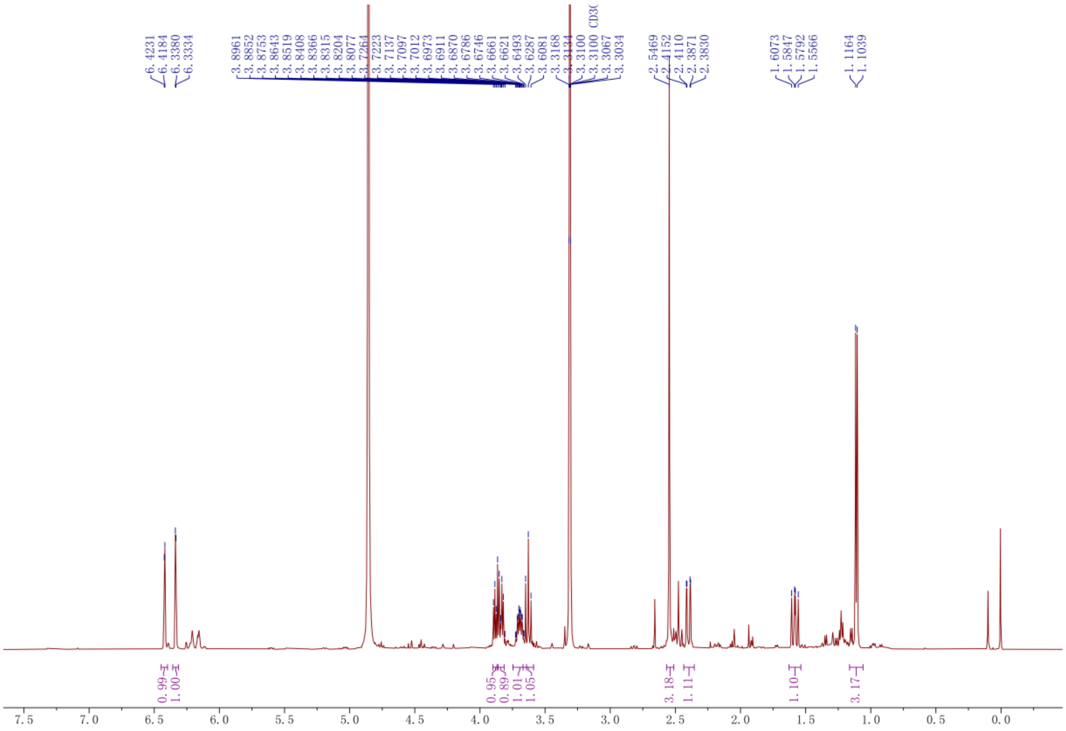

Figure S5-1. ^{1}H NMR spectrum of compound **2** (CD_3OD, 500 MHz)

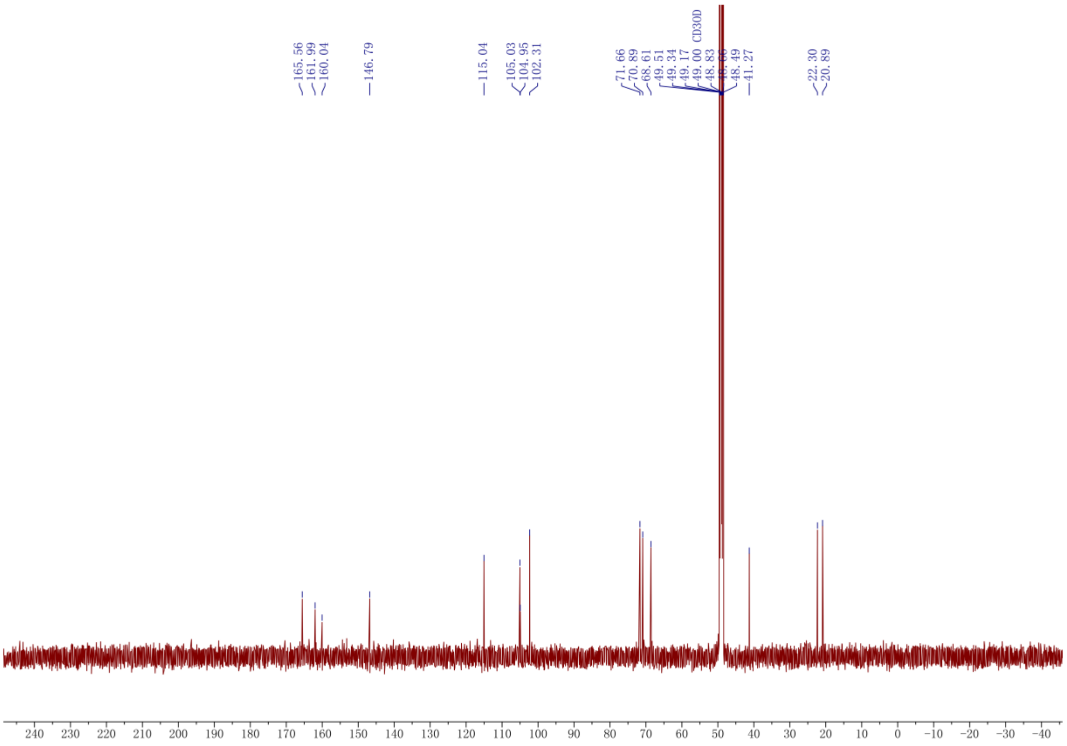

Figure S5-2. ^{13}C NMR spectrum of compound **2** (CD_3OD, 125 MHz)

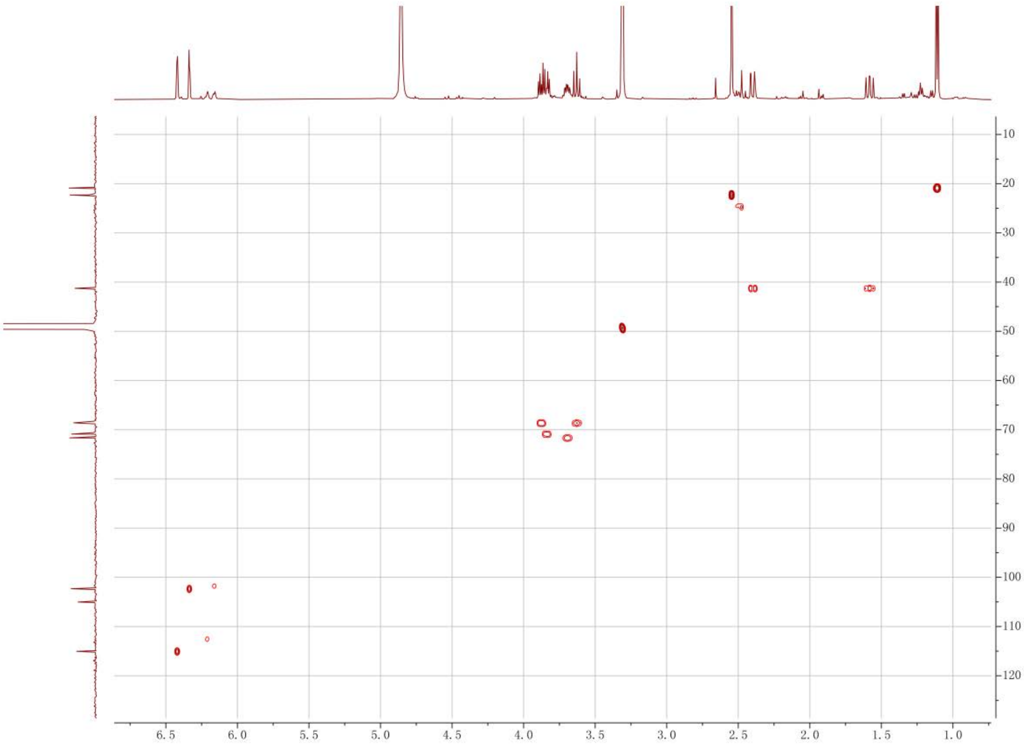

Figure S5-3. HSQC spectrum of compound **2**

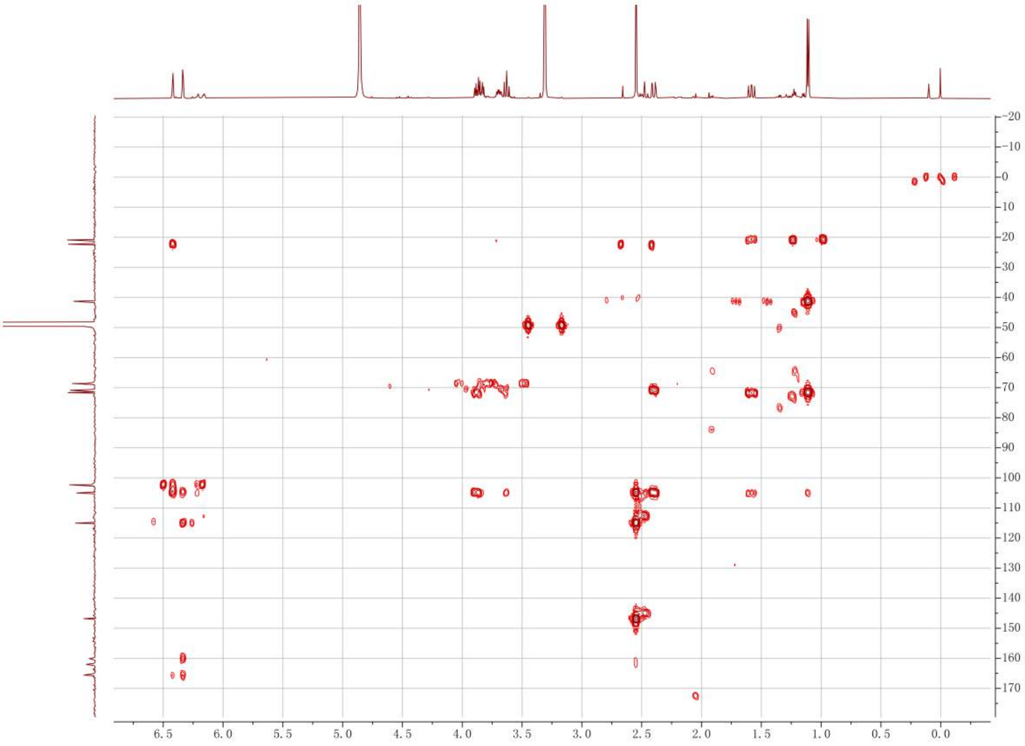

Figure S5-4. HMBC spectrum of compound **2**

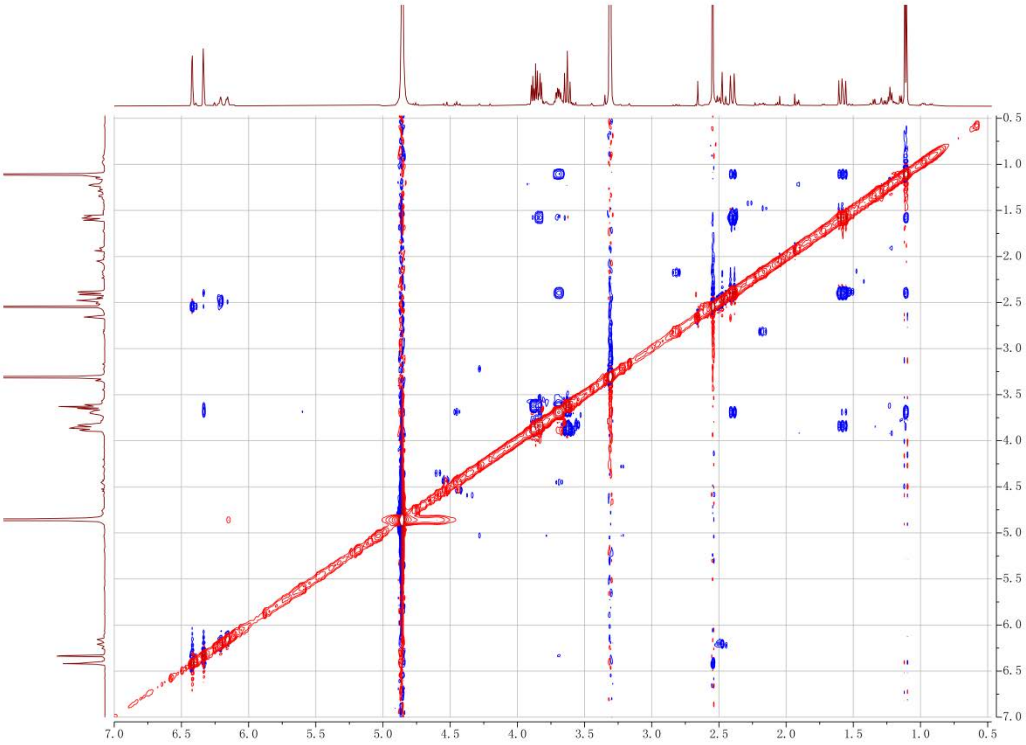

Figure S5-5. NOESY spectrum of compound **2**

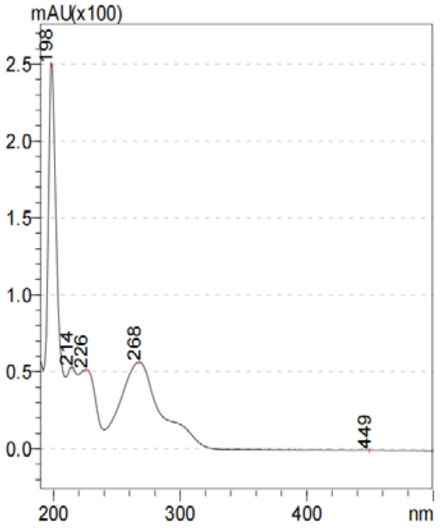

Figure S5-6. UV spectrum of compound **2**

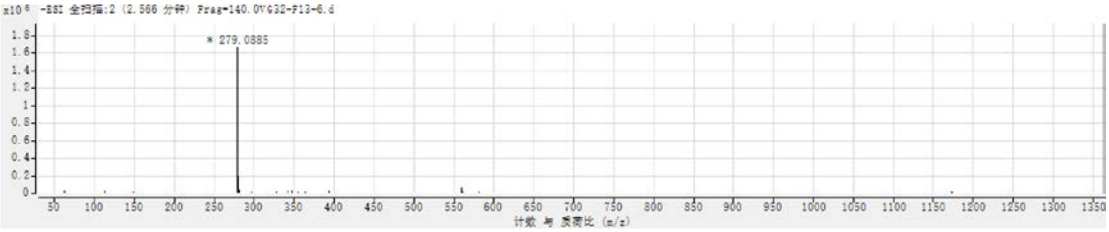

Figure S5-7. HR-ESI-MS spectrum of compound **2**

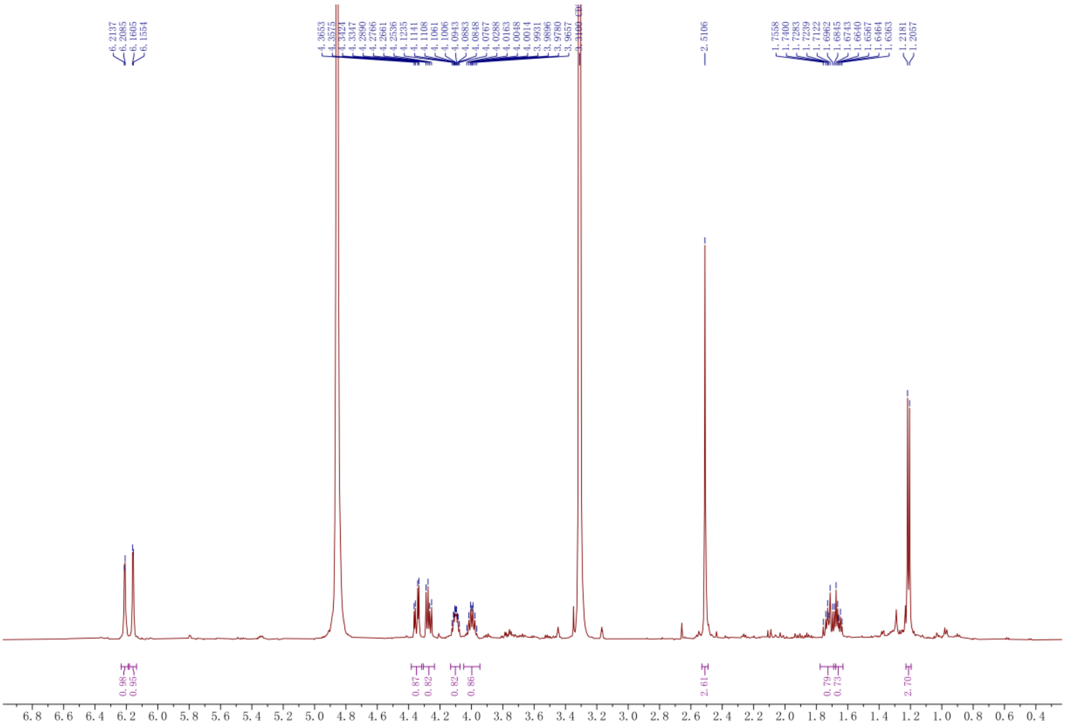

Figure S6-1. ^{1}H NMR spectrum of compound **6** (CD_3OD, 500 MHz)

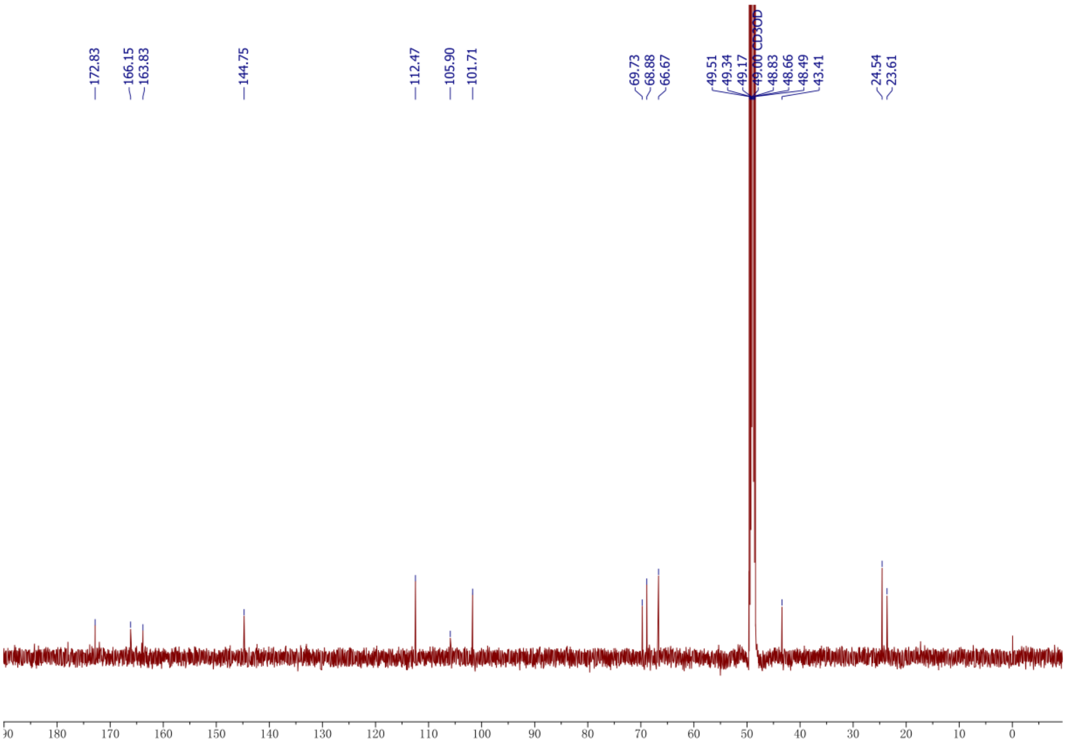

Figure S6-2. ^{13}C NMR spectrum of compound **6** (CD_3OD, 125 MHz)

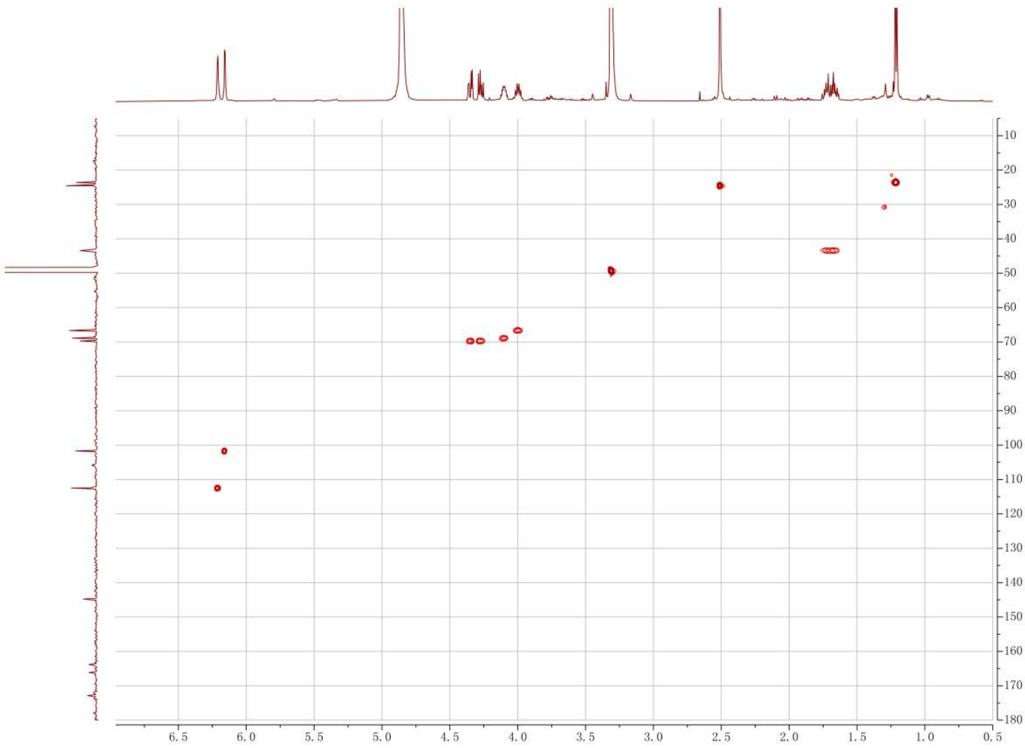

Figure S6-3. HSQC spectrum of compound **6**

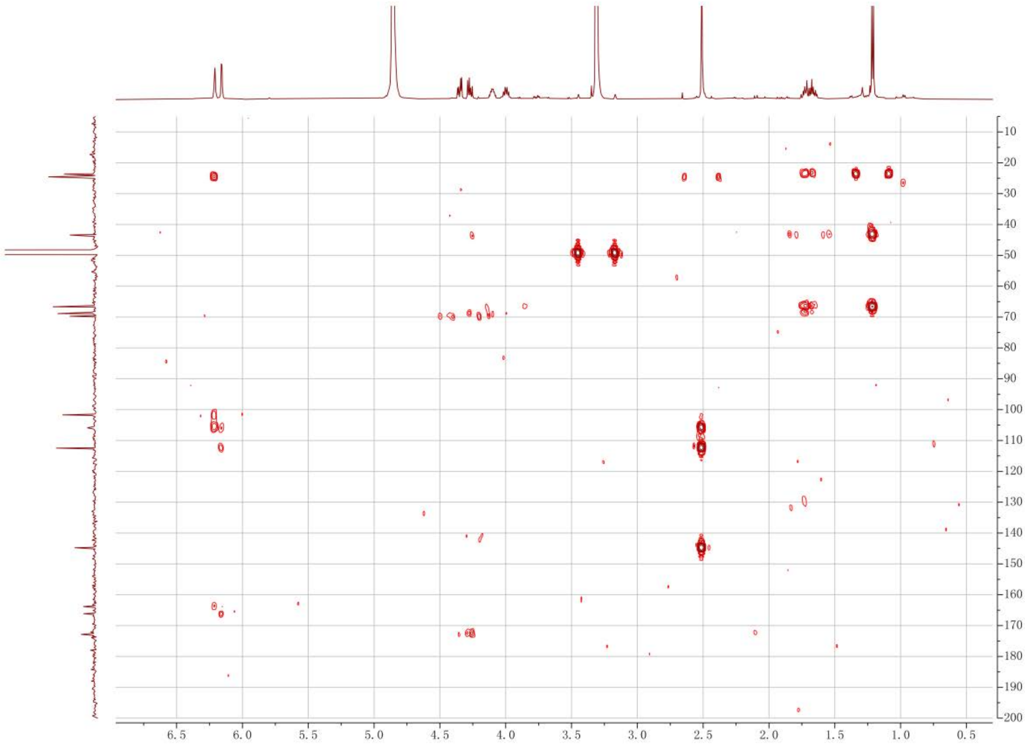

Figure S6-4. HMBC spectrum of compound **6**

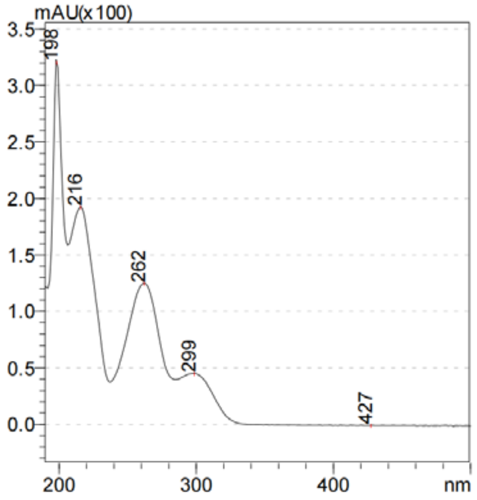

Figure S6-5. UV spectrum of compound **6**

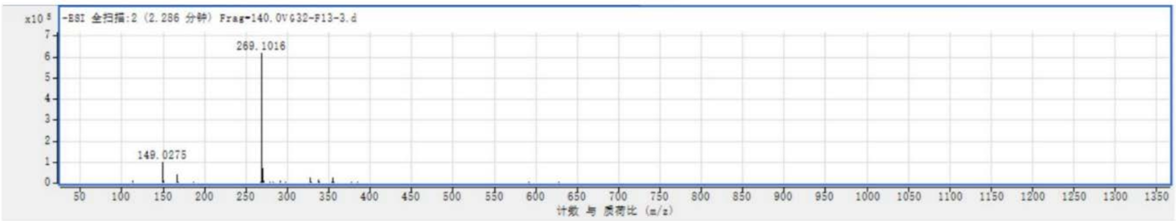

Figure S6-6. HR-ESI-MS spectrum of compound **6**

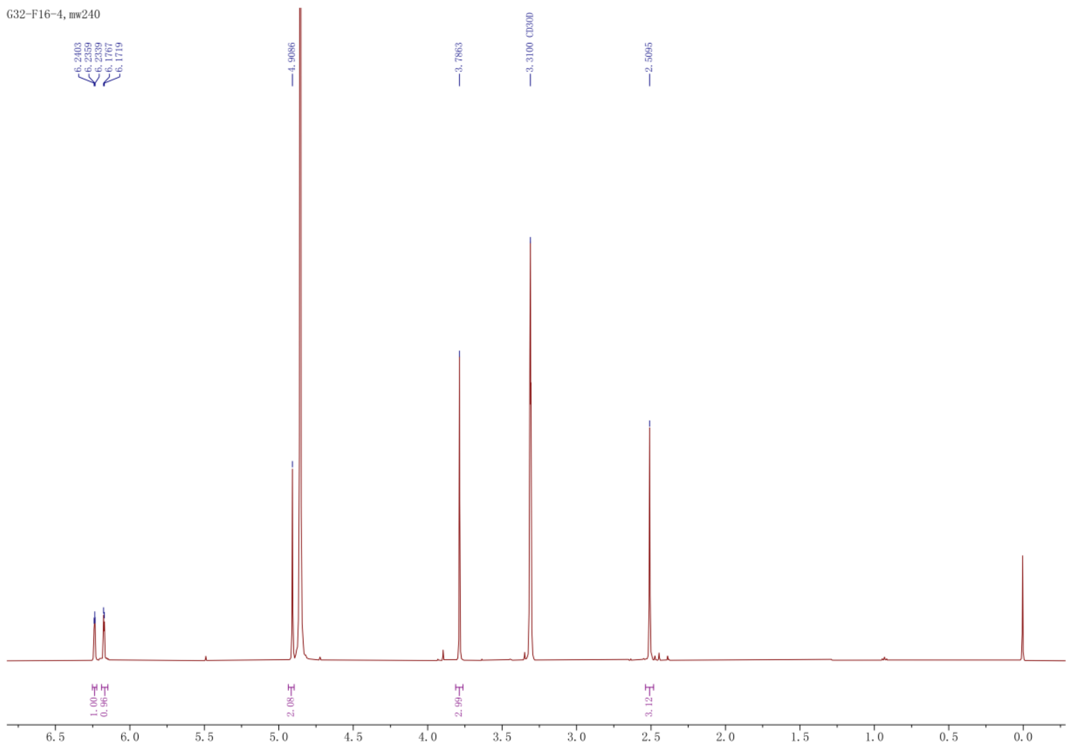

Figure S7-1. ^{1}H NMR spectrum of compound **8** (CD_3OD, 500 MHz)

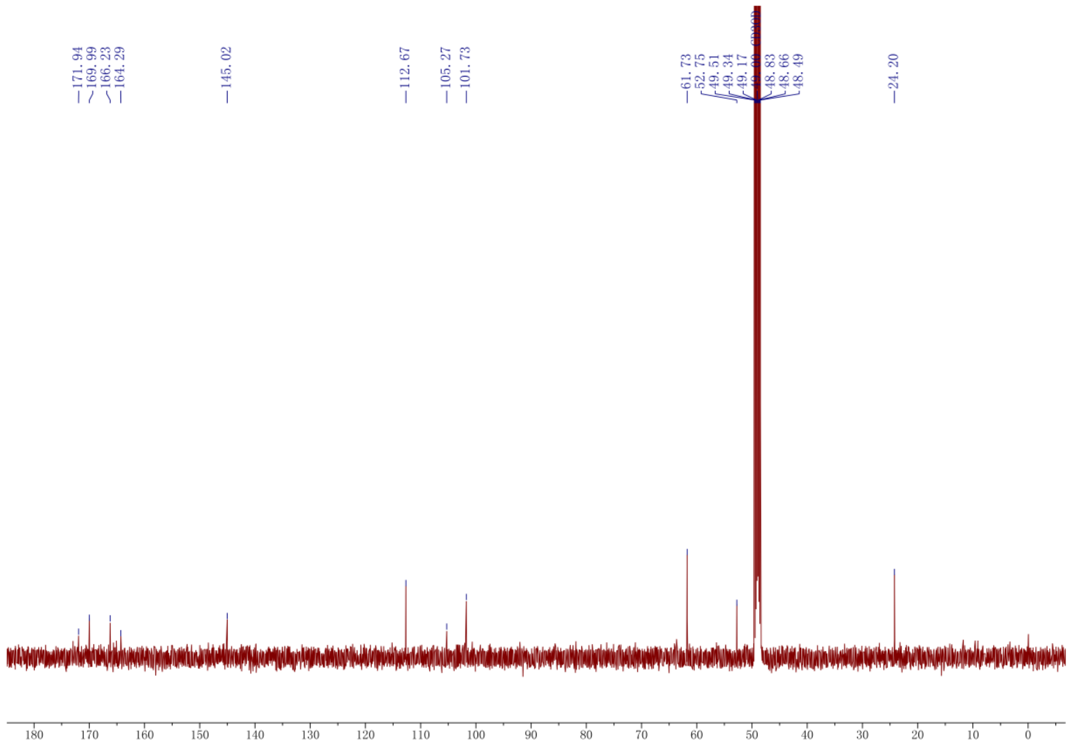

Figure S7-2. ^{13}C NMR spectrum of compound **8** (CD_3OD, 125 MHz)

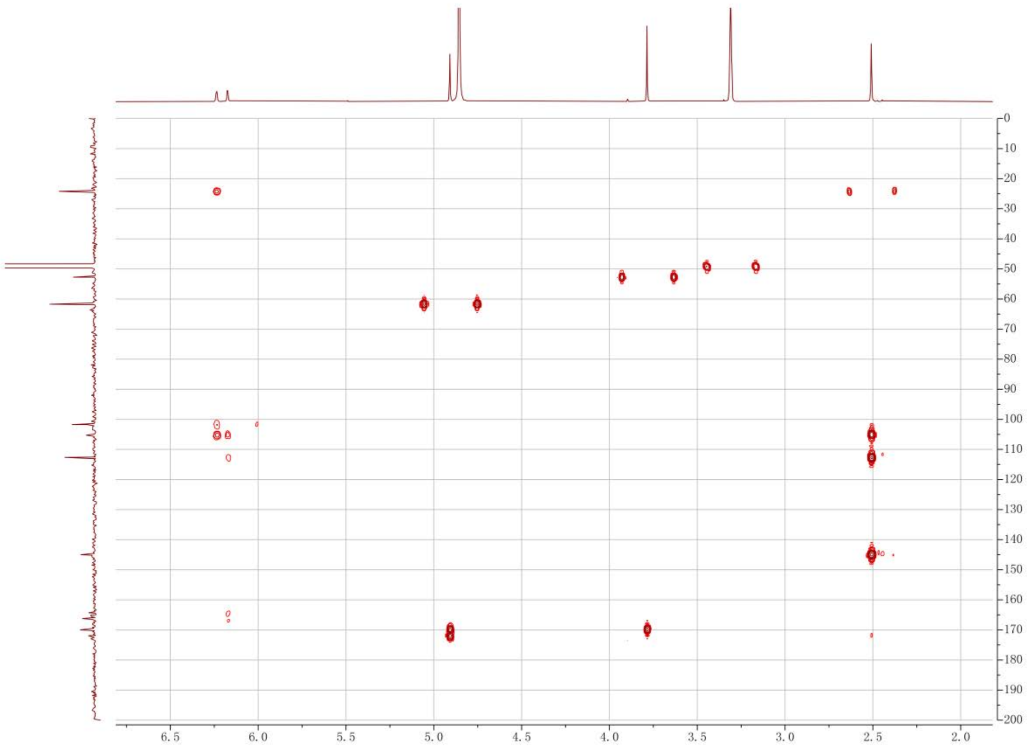

Figure S7-3. HMBC spectrum of compound **8**

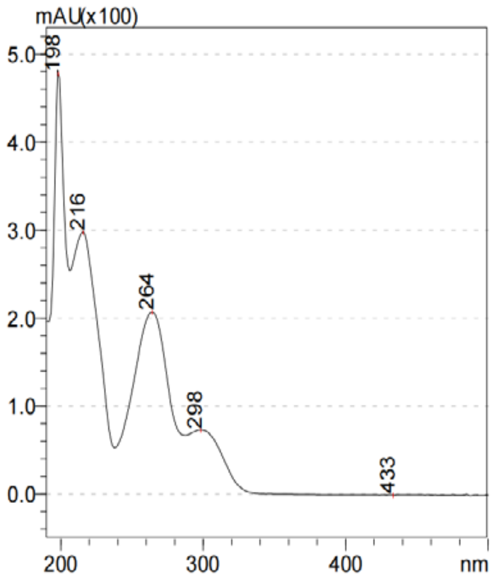

Figure S7-4. UV of compound **8**

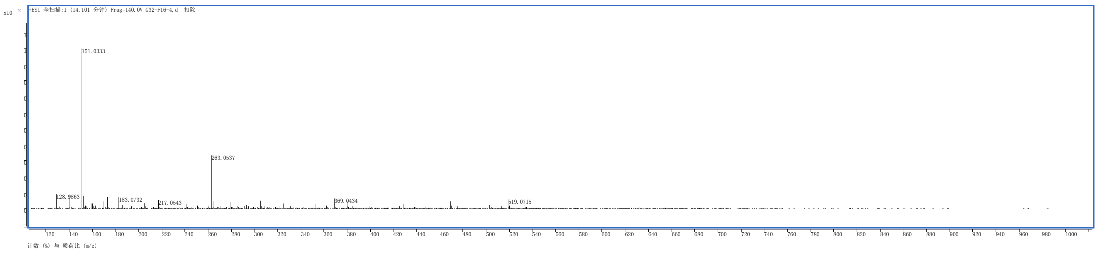

Figure S7-5. HR-ESI-MS spectrum of compound **8**
